# Supplementary material for: Microbiome-derived bile acid signatures in early life and their association with islet autoimmunity
Source: Nat Commun. 2025 Dec 3;17:38. doi: 10.1038/s41467-025-66619-6 (PMC12764907; doi:10.1038/s41467-025-66619-6)
Supplement: Supplementary file 1 — Supplementary Information [file 41467_2025_66619_MOESM1_ESM.pdf]

Supplementary information

Supplementary Figure S1. Trajectory of HDCA-conjugates during early life.

Supplementary Figure S2. Microbial production of UDCA-Asn.

Supplementary Figure S3. Manual gating strategy for identifying the CD14+ monocyte population among whole blood white blood cells analyzed by flow cytometry.

Supplementary Tables S1-S4. p-values, regression coefficients, linear mixed-effect models.

Supplementary Tables S5 Correlation microbe and microbially conjugated bile-acids

Supplementary Table S6. Output from the microbeMASST.

Supplementary Table S7. Spearman correlation microbe and MCBAs adjusted p values

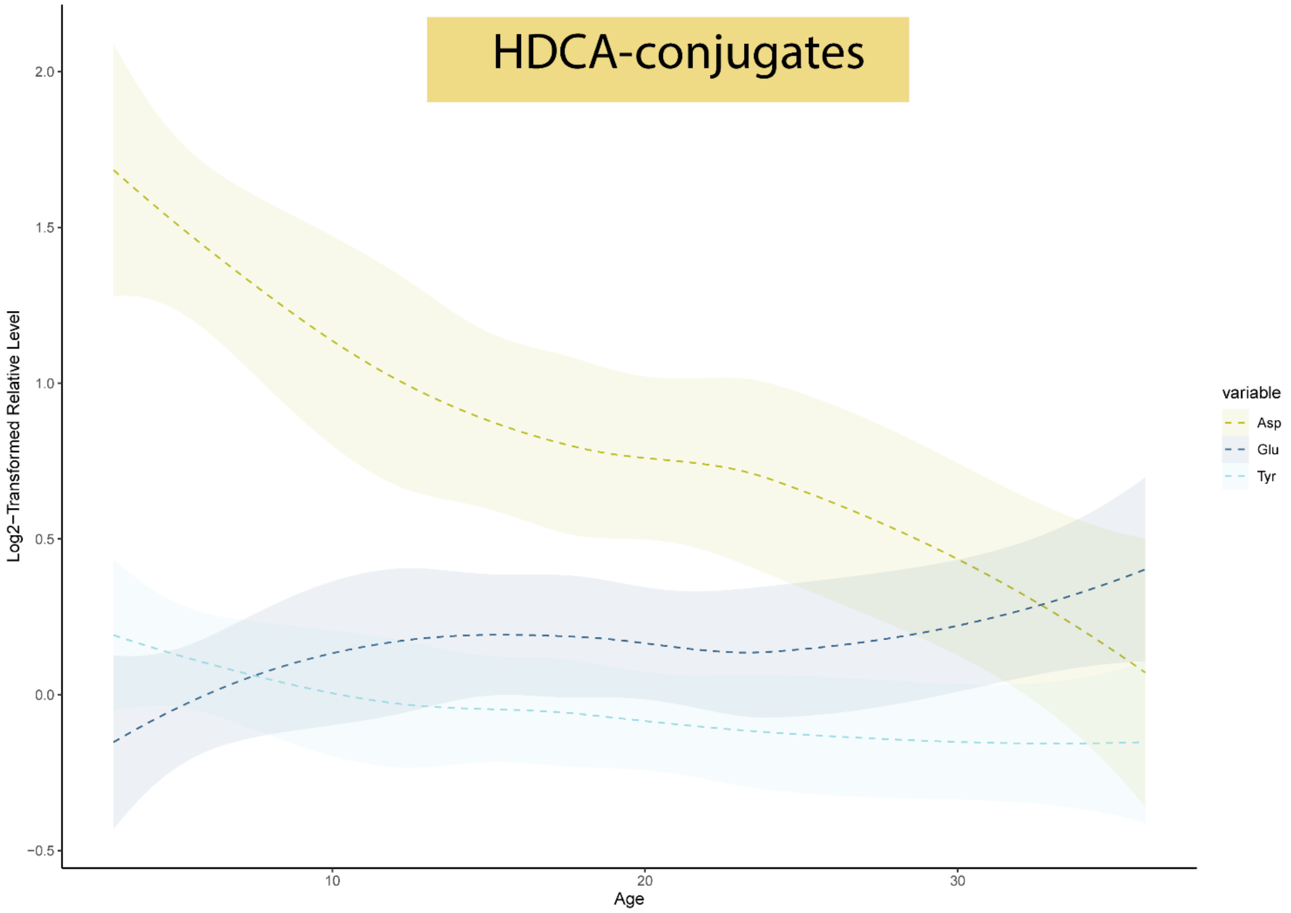

Supplementary Figure S1 Trajectories of HDCA conjugated MCBAs in early life. The loess curve plot of MCBAs over time (n = 303, fecal samples over 3, 6, 12, 18, 24 and 36 months)

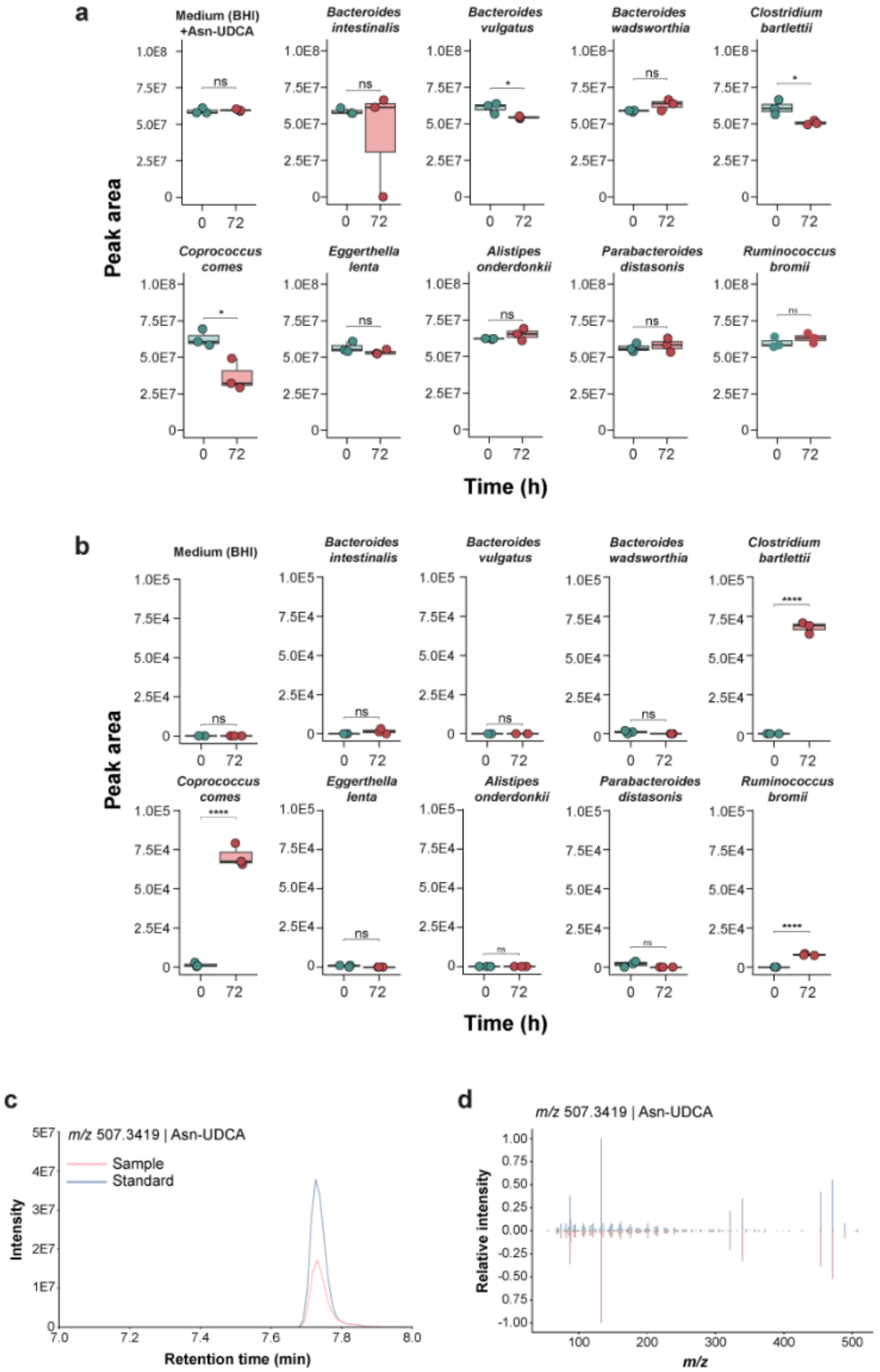

Supplementary Figure S2. Microbial production of UDCA-Asn. a. Nine gut microbial strains were cultured in the presence of 200 µM of Asn-UDCA conjugates to assess their deconjugation potential in three biological replicates. b. Asn and UDCA were added to the culture medium at 200 µM to evaluate the formation of Asn-UDCA. c. The retention time (min) was compared between the authentic standard and the biological samples. d. Mirror plot displays the MS/MS spectrum similarity between the authentic standard and the biological sample.

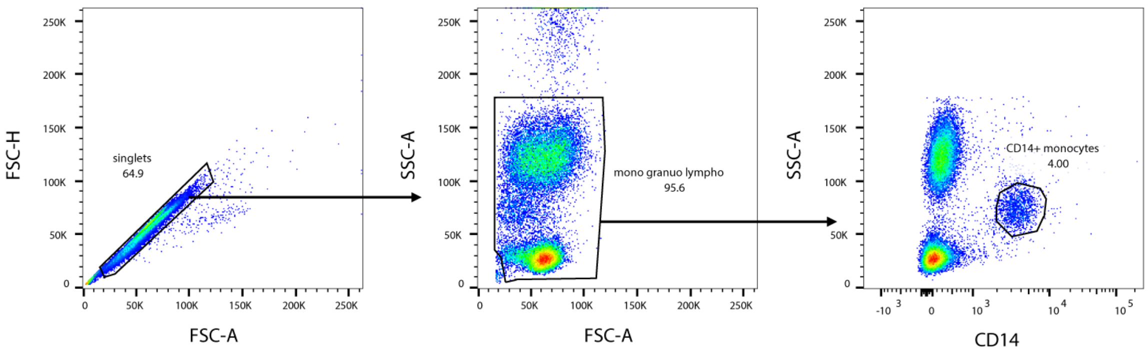

Supplementary Figure S3: Manual gating strategy for identifying the CD14+ monocyte population among whole blood white blood cells analyzed by flow cytometry.

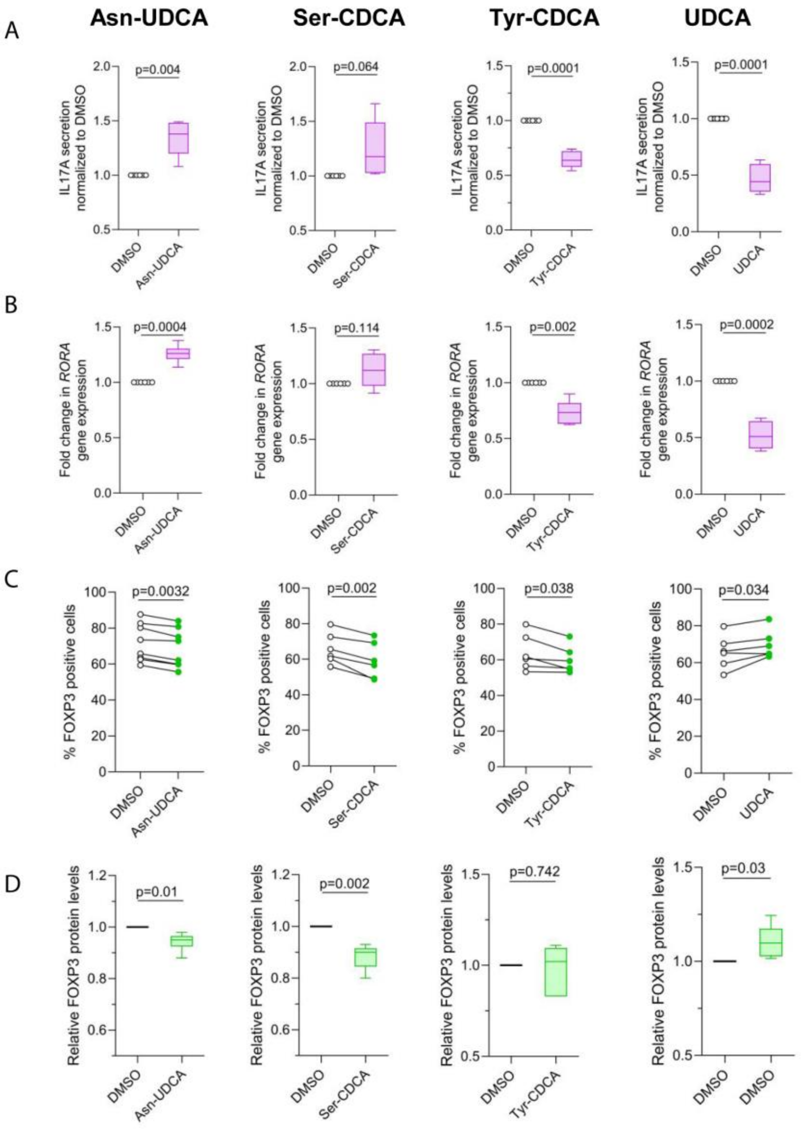

Supplementary Figure S4. Levels of IL-17A secretion in Th17 cells treated with corresponding MCBAs (upper panel), IL-17A levels normalized to DMSO control (A) and RORA gene expression (B). B) Percentage of Foxp3 positive cells (C) % FOXP3 and relative protein levels (D). Statistical significance was determined by paired t-test using six biological replicates.

Supplementary Tables S1. Age linked p-values, regression coefficients, linear mixed-effect models.

feature	metadata	coef	stderr	N	pval	qval
CAArg	AgeR	-0,34	0,113757	242	0,00321	0,035354
CAAsp	AgeR	-0,31	0,140155	242	0,02903	0,236529
CACitrulline	AgeR	-0,28	0,124528	242	0,02791	0,2317
CAGln	AgeR	-0,76	0,14781	242	5,66E-07	1,31E-05
CAGlu	AgeR	-0,95	0,158402	242	8,81E-09	2,59E-07
CAHis	AgeR	-1	0,148694	242	1,69E-10	8,26E-09
CAIle	AgeR	-0,6	0,148866	242	8,61E-05	0,001263
CALeu	AgeR	-0,61	0,149406	242	5,80E-05	0,000912
CALys	AgeR	-0,97	0,1709	242	4,68E-08	1,21E-06
CAPhe	AgeR	-0,73	0,178937	242	6,47E-05	0,000981
CAThr	AgeR	-1,03	0,140073	242	5,18E-12	2,85E-10
CAVal	AgeR	-0,52	0,120334	242	2,21E-05	0,000374
CDCAAla	AgeR	-0,36	0,101277	242	0,00041	0,005373
CDCAGln	AgeR	-0,5	0,13844	242	0,00041	0,005373
CDCAGlu	AgeR	-0,32	0,141344	242	0,02513	0,221158
CDCAIle	AgeR	-0,66	0,132857	242	1,23E-06	2,47E-05
CDCALeu	AgeR	-0,63	0,131566	242	3,61E-06	6,35E-05
CDCALys	AgeR	-0,42	0,112818	242	0,00023	0,003294
CDCAPhe	AgeR	-0,76	0,152407	242	1,34E-06	2,56E-05
CDCAPro	AgeR	1,038	0,202579	242	6,47E-07	1,42E-05
CDCAThr	AgeR	-0,42	0,142252	242	0,00385	0,041284
CDCAVal	AgeR	-0,44	0,123658	242	0,00042	0,005373
DCAAla	AgeR	0,437	0,103587	242	3,60E-05	0,000586
DCAArg	AgeR	0,675	0,10919	242	2,98E-09	1,03E-07
DCAAsn	AgeR	0,163	0,0481	242	0,00084	0,010269
DCAAsp	AgeR	0,855	0,103331	242	1,32E-14	1,94E-12
DCACitrulline	AgeR	0,687	0,117537	242	1,84E-08	5,05E-07
DCAGln	AgeR	0,323	0,106501	242	0,00267	0,030155
DCAGlu	AgeR	0,497	0,102583	242	2,41E-06	4,42E-05
DCAHis	AgeR	0,308	0,061588	242	1,17E-06	2,45E-05
DCAIle	AgeR	1,289	0,131373	242	6,10E-19	1,34E-16
DCALeu	AgeR	1,289	0,131373	242	6,10E-19	1,34E-16
DCALys	AgeR	0,827	0,108557	242	7,85E-13	4,93E-11
DCAMet	AgeR	1,016	0,127095	242	7,90E-14	5,80E-12
DCAPhe	AgeR	0,668	0,10808	242	3,04E-09	1,03E-07
DCAPro	AgeR	1,226	0,195711	242	1,76E-09	7,03E-08
DCASer	AgeR	0,514	0,09605	242	2,20E-07	5,37E-06
DCAThr	AgeR	0,939	0,147987	242	1,27E-09	5,57E-08
DCATrp	AgeR	0,96	0,119507	242	6,04E-14	5,32E-12

DCAVal	AgeR	0,902	0,109708	242	1,88E-14	2,06E-12
HDCAAsp	AgeR	-0,48	0,078315	242	5,66E-09	1,78E-07
HDCAGlu	AgeR	0,124	0,060285	242	0,04118	0,29141
UDCAAsp	AgeR	0,274	0,084702	242	0,00144	0,016731
UDCACitrulline	AgeR	0,158	0,05754	242	0,00664	0,067976
UDCAGlu	AgeR	0,38	0,117298	242	0,0014	0,016595
UDCALys	AgeR	0,086	0,042211	242	0,04365	0,29141
UDCAThr	AgeR	0,429	0,122135	242	0,00054	0,006804
UDCAVal	AgeR	0,297	0,117186	242	0,01193	0,114157

Supplementary Tables S2. p-values, regression coefficients, linear mixed-effect models.

feature	metadata	value	coef	stderr	N	pval	qval
CDCATyr	Case	OneAb	0,782438	0,333313	242	0,02282	0,213635
DCAVal	Case	OneAb	-0,71687	0,314617	242	0,02657	0,229228
CDCAAla	Case	TwoAb	-0,72806	0,322955	242	0,02789	0,2317
DCAIle	Case	OneAb	-0,88972	0,402221	242	0,031346	0,246287
DCALeu	Case	OneAb	-0,88972	0,402221	242	0,031346	0,246287
CDCASer	Case	TwoAb	-0,55837	0,269143	242	0,042827	0,29141
DCAPro	Case	OneAb	-0,90466	0,44566	242	0,043485	0,29141
UDCAAsn	Case	TwoAb	-0,43593	0,211612	242	0,043905	0,29141
CACys	Case	TwoAb	0,095334	0,047306	242	0,04501	0,29141
CACitrulline	Case	TwoAb	-1,03861	0,508	242	0,045579	0,29141

Supplementary Tables S3. p-values, regression coefficients, linear mixed-effect models.

feature	metadata	coef	stderr	N	pval	qval
UDCAVal	Sex	-0,39137	0,133611	242	0,0051	0,05343
HDCACitrulline	Sex	0,063367	0,02413	242	0,009202	0,089974
CDCAPhe	Sex	-0,51362	0,221786	242	0,024455	0,219596
CAHis	Sex	-0,50153	0,238037	242	0,040015	0,29141
UDCAGlu	Sex	-0,31945	0,152452	242	0,040873	0,29141
CAIle	Sex	-0,43499	0,20813	242	0,041229	0,29141
CALeu	Sex	-0,42831	0,208436	242	0,044627	0,29141
UDCATyr	Sex	-0,23384	0,1142	242	0,045698	0,29141
UDCAPhe	Sex	-0,28427	0,141287	242	0,049261	0,309639

Supplementary Tables S4. Breast feeding length related p-values, regression coefficients, linear mixed-effect models.

feature	metadata	coef	stderr	N	pval	qval
CDCAAla	bflength	0,324336	0,117051	242	0,008077	0,080773
CAPro	bflength	0,076943	0,032909	242	0,023801	0,218176
CDCATyr	bflength	0,352204	0,16874	242	0,042498	0,29141

Supplementary Tables S5. Correlation microbe and microbially conjugated bile-acids

Microbes	CDCA Ala	CDCA Ser	UDCA Asn	CA Cys	CACitrulline	CDCA Tyr	DCA val	DCA IsoLeu	DCA Lue	DCA Pro
s__Roseburia_intestinalist__Roseburia_intestinalis_unclassified	0,104035	-0,0093	-0,00981	0,119181	-0,11929	0,050585	0,180174	0,172905	0,172905	0,418507
s__Bilophila_wadsworthiat__GCF_000185705	-0,07605	0,006685	-0,11617	0,259184	-0,08847	-0,17121	0,168112	0,213191	0,213191	0,347807
s__Faecalibacterium_prausnitziit__Faecalibacterium_prausnitzii_unclassified	-0,1476	0,220198	-0,16146	0,065456	-0,01392	0,100525	0,259099	0,22579	0,22579	0,338589
s__Lachnospiraceae_bacterium_5_1_63FAAt__GCF_000185525	-0,24788	0,103232	-0,17612	0,093483	0,060957	0,009855	0,234894	0,27151	0,27151	0,333764
s__Ruminococcus_obeumt__Ruminococcus_obeum_unclassified	-0,26073	-0,03011	0,002266	0,090677	-0,02296	-0,13982	0,301125	0,394264	0,394264	0,325983
s__Anaerostipes_hadrust__GCF_000332875	-0,16682	-0,07072	0,030766	0,128174	0,064772	-0,04237	0,090136	0,153588	0,153588	0,308864
s__Eubacterium_rectalet__Eubacterium_rectale_unclassified	-0,29848	0,153118	0,052772	0,062639	0,07991	0,095507	0,191651	0,243286	0,243286	0,306329
s__Coprococcus_comest__GCF_000155875	-0,09722	-0,01149	-0,03541	0,273492	-0,10142	-0,16383	0,115894	0,235663	0,235663	0,283158
s__Bacteroides_uniformist__Bacteroides_uniformis_unclassified	-0,02424	0,061504	-0,09661	0,020733	0,118052	0,138478	0,268698	0,286351	0,286351	0,248152
s__Bacteroides_faecist__GCF_000226135	-0,09306	0,097629	0,061508	0,235134	-0,06309	0,01024	0,196902	0,220003	0,220003	0,239369
s__Roseburia_inulinivoranst__GCF_000174195	-0,21343	0,10239	-0,0498	0,067897	0,086329	0,040816	0,301825	0,333307	0,333307	0,239198

s__Clostridium_hathewayit__Clostridium_hathewayi_unclassified	-0,06477	0,044857	-0,05823	-0,07495	-0,00259	0,066856	-0,12527	-0,1137	-0,1137	0,238156
s__Eggerthella_lentat__GCF_000024265	-0,09829	0,051238	0,023809	0,118701	-0,17896	0,042703	-0,09095	-0,09818	-0,09818	0,237181
s__Roseburia_hominist__GCF_000225345	-0,20913	-0,01922	-0,09089	0,159583	-0,01957	-0,05673	0,244035	0,315509	0,315509	0,234305
s__Prevotella_buccaet__Prevotella_buccae_unclassified	0,067946	-0,08981	0,147107	-0,01604	-0,0151	0,058237	-0,02218	-0,04899	-0,04899	0,226691
s__Dorea_longicatenat__GCF_000154065	-0,09829	0,016627	0,025394	-0,05265	-0,12496	-0,06698	0,14287	0,211055	0,211055	0,225964
s__Ruminococcus_lactarist__Ruminococcus_lactaris_unclassified	-0,10017	-0,10983	0,062607	-0,0488	0,031844	-0,04281	0,192894	0,246307	0,246307	0,218081
s__Eubacterium_eligenst__GCF_000146185	-0,16338	-0,016	0,075337	-0,07248	-0,09037	-0,06679	0,413277	0,432467	0,432467	0,215048
s__Lachnospiraceae_bacterium_3_1_46FAAt__GCF_000209405	-0,0794	0,169407	0,016106	-0,04351	-0,00976	0,122468	0,105389	0,075922	0,075922	0,201423
s__Collinsella_aerofacienst__GCF_000169035	-0,00852	-0,06764	-0,08604	0,180666	0,13873	-0,09541	0,233292	0,293787	0,293787	0,198491
s__Desulfovibrio_pigert__GCF_000156375	-0,06181	0,103778	-0,04528	-0,01303	-0,10298	-0,1296	0,179301	0,18223	0,18223	0,193993
s__Clostridium_bartlettiit__GCF_000154445	-0,00637	-0,02455	0,064644	0,094422	0,02843	-0,03585	-0,02035	0,008207	0,008207	0,193843
s__Bacteroidales_bacterium_ph8t__GCF_000311925	-0,07871	-0,02614	-0,1107	-0,03187	-0,14153	-0,13464	0,281226	0,24266	0,24266	0,192587
s__Eubacterium_ramulust__GCF_000469345	-0,0	-0,1	-0,0992	-0,0	-0,1776	-0,2	0,273818	0,30437	0,30437	0,187874

	3858	5993		2856		1745				
s__Bacteroides_finegoldiit__Bacteroides_finegoldii_unclassified	0,030475	0,088996	-0,07258	-0,02089	0,170851	0,060849	0,158368	0,12235	0,12235	0,187326
s__Eubacterium_cylindroidest__GCF_000469305	-0,07605	-0,08981	-0,05571	0,571964	-0,1267	-0,15945	0,041691	0,169688	0,169688	0,186329
s__Ruminococcus_bromiit__GCF_000209875	-0,13525	-0,0643	-0,04889	0,070286	-0,03476	-0,07331	0,321789	0,32848	0,32848	0,178594
s__Ruminococcus_callidust__GCF_000468015	-0,15113	0,046432	-0,02088	-0,03187	-0,03685	-0,05371	0,147445	0,215759	0,215759	0,177589
s__Clostridium_bolteaet__Clostridium_bolteae_unclassified	-0,0229	0,020473	0,118358	0,054122	-0,01943	0,062966	0,098683	0,068827	0,068827	0,174969
s__Parasutterella_excrementihominist__GCF_000205025	-0,05143	-0,11047	-0,06436	-0,04076	-0,01972	-0,00533	0,038081	0,075452	0,075452	0,169076
s__Burkholderiales_bacterium_1_1_47t__GCF_000144975	-0,03962	-0,08943	-0,13174	-0,03792	-0,02429	0,009727	0,080769	0,092747	0,092747	0,166618
s__Odoribacter_splanchnicust__GCF_000190535	-0,15113	-0,02389	0,001214	-0,03187	-0,05167	-0,11182	0,244133	0,253117	0,253117	0,162964
s__Collinsella_tanakaeit__GCF_000225705	-0,04351	-0,05138	-0,03187	-0,00917	0,136336	-0,09122	0,202818	0,189426	0,189426	0,162031
s__Bacteroides_finegoldiit__GCF_000156195	-0,04351	-0,05138	-0,03187	-0,00917	0,136336	-0,09122	0,202818	0,189426	0,189426	0,162031
s__Clostridium_hiranonist__GCF_000156055	-0,04351	-0,05138	-0,03187	-0,00917	0,136336	-0,09122	0,202818	0,189426	0,189426	0,162031
s__Bacteroides_stercorist__GCF_000154525	-0,04351	-0,05138	-0,03187	-0,00917	0,136336	-0,09122	0,202818	0,189426	0,189426	0,162031

s__Parabacteroides_goldsteiniit__Parabacteroides_goldsteinii_unclassified	-0,08822	-0,10418	-0,06462	0,479014	-0,05595	-0,11101	0,164705	0,207022	0,207022	0,160727
s__Lactococcus_lactist__Lactococcus_lactis_unclassified	-0,01382	0,021829	0,03076	-0,03936	0,060449	-0,0008	0,075747	0,059656	0,059656	0,154878
s__Eubacterium_biformet__GCF_000156655	0,056708	0,024245	-0,06462	-0,0186	0,086894	-0,0676	0,19945	0,190028	0,190028	0,153909
s__Sutterella_wadsworthensist__GCF_000411515	-0,06181	-0,07299	-0,04528	0,710343	-0,10298	-0,1296	0,096127	0,171995	0,171995	0,152981
s__Alloprevotella_tanneraet__GCF_000159995	-0,04351	-0,05138	-0,03187	-0,00917	-0,07248	-0,09122	-0,0614	-0,07125	-0,07125	0,149916
s__Coprococcus_catust__GCF_000210555	-0,09909	-0,11701	-0,07258	-0,02089	0,078251	0,025487	0,029695	0,046243	0,046243	0,148072
s__Alistipes_putredinist__GCF_000154465	-0,18752	-0,0732	-0,01991	0,13358	-0,07233	-0,1364	0,226327	0,271757	0,271757	0,146274
s__Dorea_formicigeneranst__Dorea_formicigenerans_unclassified	-0,1107	-0,01278	-0,17401	0,189941	0,049843	-0,09551	0,169741	0,239004	0,239004	0,145864
s__Paraprevotella_xylaniphilat__GCF_000205165	0,138435	0,094941	-0,04528	-0,01303	0,009595	0,005767	-0,08724	0,001526	0,001526	0,144375
s__Alistipes_sp_HGB5t__GCF_000183485	-0,04351	-0,05138	-0,03187	-0,00917	-0,07248	-0,09122	-0,0614	-0,07125	-0,07125	0,143859
s__Anaeroglobus_geminatust__GCF_000239275	-0,06181	-0,07299	-0,04528	-0,01303	-0,10298	0,13152	0,017543	-0,00826	-0,00826	0,143789
s__Lachnospiraceae_bacterium_7_1_58FAAt__GCF_000242155	-0,20926	0,046149	0,014527	0,104901	-0,01829	0,034918	0,059257	0,074621	0,074621	0,143555
s__Barnesiella_intestinihominist__GCF_000296465	-0,1	-0,0	0,046648	0,199237	0,05663	-0,0	0,184457	0,238549	0,238549	0,141103

	8663	4342				1877				
s__Ruminococcus_flavefacienst__Ruminococcus_flavefaciens_unclassified	-0,04351	-0,05138	-0,03187	-0,00917	0,129433	0,036809	0,146997	0,116436	0,116436	0,14083
s__Fusobacterium_mortiferumt__GCF_000158195	-0,06181	-0,07299	-0,04528	-0,01303	0,056157	0,021352	0,198214	0,197538	0,197538	0,13841
s__Eubacterium_siraeumt__Eubacterium_siraeum_unclassified	-0,10426	-0,07073	0,132031	-0,03792	-0,16345	-0,10812	0,120804	0,162357	0,162357	0,138316
s__Clostridium_nexilet__GCF_000156035	0,01091	0,238875	-0,15623	-0,06633	0,115778	0,160378	-0,12051	-0,14182	-0,14182	0,137634
s__Bacteroides_salyersiaet__Bacteroides_salyersiae_unclassified	0,027896	-0,03062	-0,07258	0,422064	-0,10769	-0,14652	0,171466	0,213799	0,213799	0,136284
s__Bacteroides_clarust__GCF_000195615	-0,04568	-0,15008	-0,09309	-0,0268	-0,02389	0,143669	0,164174	0,130166	0,130166	0,136254
s__Eubacterium_halliit__GCF_000173975	-0,17064	-0,10213	-0,01015	-0,07371	-0,03554	-0,16784	0,200971	0,257818	0,257818	0,13226
s__Porphyromonas_someraet__GCF_000372405	-0,04351	-0,05138	-0,03187	-0,00917	-0,07248	-0,09122	0,139554	0,126863	0,126863	0,131745
s__Sutterella_wadsworthensist__GCF_000297775	-0,04351	-0,05138	-0,03187	-0,00917	-0,07248	-0,09122	0,139554	0,126863	0,126863	0,131745
s__Haemophilus_pittmaniaet__GCF_000223275	-0,04351	-0,05138	-0,03187	-0,00917	-0,07248	0,120028	0,087454	0,060825	0,060825	0,128716
s__Streptococcus_lutetiensist__GCF_000441535	-0,14342	0,124951	-0,10506	-0,03024	-0,02595	0,08823	-0,14753	-0,10842	-0,10842	0,127705
s__Lactobacillus_ruminist__Lactobacillus_ruminis_unclassified	0,078522	0,130778	-0,05571	-0,01604	-0,1267	0,108353	-0,10733	-0,04207	-0,04207	0,125337

s__Campylobacter_gracilist__GCF_000175875	-0,06181	-0,07299	-0,04528	-0,01303	-0,10298	-0,0192	0,080819	0,055731	0,055731	0,124954
s__Prevotella_oralist__Prevotella_oralis_unclassified	0,199214	-0,05138	0,309999	-0,00917	0,115627	0,072017	-0,0614	-0,07125	-0,07125	0,122659
s__Bacteroides_plebeiust__GCF_000187895	0,05418	0,078164	-0,09309	-0,0268	0,029669	-0,17423	0,063014	0,032963	0,032963	0,120053
s__Clostridium_symbiosumt__Clostridium_symbiosum_unclassified	-0,13167	0,17639	-0,02815	-0,06879	-0,02764	0,146134	0,041636	0,031524	0,031524	0,117
s__Butyricicoccus_pullicaecorumt__GCF_000398925	0,040626	0,015399	0,092471	-0,0186	-0,05592	0,10393	-0,04699	-0,0757	-0,0757	0,11587
s__Bacteroides_fragilist__Bacteroides_fragilis_unclassified	-0,26205	-0,13363	-0,14205	0,017543	-0,08273	-0,04987	0,255742	0,28266	0,28266	0,113953
s__Eubacterium_ventriosumt__GCF_000153885	-0,08652	-0,14874	-0,12149	-0,03497	-0,14083	-0,22721	0,22979	0,231702	0,231702	0,110633
s__Leuconostoc_pseudomesenteroidest__Leuconostoc_pseudomesenteroides_unclassified	-0,04351	0,12947	-0,03187	-0,00917	-0,07248	0,094422	-0,0614	-0,07125	-0,07125	0,110544
s__Lactococcus_phage_936_sensu_latot__PRJNA17757	-0,04351	0,12947	-0,03187	-0,00917	-0,07248	0,094422	-0,0614	-0,07125	-0,07125	0,110544
s__Lactococcus_phage_phi7t__PRJNA213073	-0,04351	0,12947	-0,03187	-0,00917	-0,07248	0,094422	-0,0614	-0,07125	-0,07125	0,110544
s__Haemophilus_parainfluenzaet__Haemophilus_parainfluenzae_unclassified	0,167343	0,053426	0,179301	0,124011	-0,11342	0,111864	-0,02579	-0,06961	-0,06961	0,109054
s__Clostridium_clostridioformet__Clostridium_clostridioforme_unclassified	-0,2102	0,141024	-0,01011	-0,05768	-0,04016	0,088911	0,069951	0,025528	0,025528	0,107997
s__Bacteroides_coprophilust__GCF_000157915	-0,0	-0,0	-0,0	-0,0	-0,07248	-0,0	0,161882	0,165096	0,165096	0,107516

	4351	5138	3187	0917		9122				
s__Catenibacterium_mitsuokait__GCF_000173795	-0,04351	-0,05138	-0,03187	-0,00917	-0,07248	-0,09122	0,161882	0,165096	0,165096	0,107516
s__Bacteroides_ovatust__Bacteroides_ovatus_unclassified	-0,05799	-0,05992	-0,05528	0,071578	0,052312	0,110611	0,245847	0,213183	0,213183	0,105584
s__Actinomyces_johnsoniit__Actinomyces_johnsonii_unclassified	-0,04351	-0,05138	-0,03187	-0,00917	-0,07248	-0,09122	-0,0614	0,050398	0,050398	0,104487
s__Prevotella_stercoreat__GCF_000235885	-0,04351	-0,05138	-0,03187	-0,00917	-0,07248	-0,09122	-0,0614	0,050398	0,050398	0,104487
s__Lactobacillus_delbrueckiit__Lactobacillus_delbrueckii_unclassified	-0,04351	-0,05138	-0,03187	-0,00917	-0,07248	-0,09122	-0,0614	0,050398	0,050398	0,104487
s__Ruminococcus_torquest__GCF_000210035	-0,04351	-0,05138	-0,03187	-0,00917	-0,07248	-0,09122	-0,0614	0,050398	0,050398	0,104487
s__Streptococcus_phage_ALQ13_2t__PRJNA42593	-0,04351	-0,05138	-0,03187	-0,00917	-0,07248	-0,09122	-0,0614	0,050398	0,050398	0,104487
s__Haemophilus_paraphrohaemolyticust__GCF_000260675	-0,06181	0,083156	-0,04528	-0,01303	0,211054	0,202684	-0,08724	-0,10123	-0,10123	0,101426
s__Alistipes_shahiit__GCF_000210575	-0,17293	-0,0132	0,070873	0,221398	0,086336	-0,02452	0,12768	0,196774	0,196774	0,100407
s__Lachnospiraceae_bacterium_8_1_57FAAt__GCF_000185545	-0,07564	0,000905	-0,00183	-0,04351	-0,19345	-0,07092	0,085546	0,118146	0,118146	0,096681
s__Lachnospiraceae_bacterium_1_1_57FAAt__GCF_000218445	-0,10314	-0,07304	0,151542	-0,03647	-0,07498	0,066684	0,031533	-0,00309	-0,00309	0,095725
s__Alistipes_onderdonkiit__GCF_000374505	-0,26294	0,085899	-0,00387	0,090273	-0,0185	-0,07523	0,285499	0,316279	0,316279	0,09562

s__Bifidobacterium_longumt__Bifidobacterium_longum_unclassified	0,023759	-0,01381	-0,01458	-0,0287	-0,03436	0,047929	-0,03663	-0,05514	-0,05514	0,094817
s__Bacteroides_vulgatust__Bacteroides_vulgatus_unclassified	0,028288	0,098664	-0,22968	-0,04146	-0,00525	0,003448	0,090975	0,059649	0,059649	0,094043
s__Gemella_haemolysanst__Gemella_haemolysans_unclassified	0,226692	0,150021	-0,03187	-0,00917	-0,07248	0,081619	-0,0614	-0,07125	-0,07125	0,092373
s__Bifidobacterium_angulatumt__GCF_000156635	-0,06181	-0,07299	-0,04528	-0,01303	0,024574	0,047457	0,025402	0,103628	0,103628	0,091843
s__Alistipes_indistinctust__GCF_000231275	-0,11833	0,032825	-0,08668	-0,02495	-0,05263	0,066602	0,128097	0,137194	0,137194	0,090235
s__Streptococcus_pasteurianust__GCF_000270165	-0,06181	0,20456	-0,04528	-0,01303	-0,10298	0,173148	0,035471	0,00092	0,00092	0,08975
s__Bacteroides_stercorist__Bacteroides_stercoris_unclassified	0,02579	-0,08869	-0,0992	-0,02856	0,12246	0,011302	0,145129	0,126082	0,126082	0,089476
s__Streptococcus_infantariust__Streptococcus_infantarius_unclassified	-0,06181	0,077264	-0,04528	-0,01303	-0,10298	0,042476	0,035471	0,086548	0,086548	0,085487
s__Akkermansia_muciniphilat__GCF_000020225	-0,26587	0,113263	0,063316	-0,08114	0,118879	-0,03958	0,193492	0,214651	0,214651	0,085218
s__Pseudoflavonifractor_capillosust__GCF_000169255	-0,06181	0,086129	-0,04528	-0,01303	0,017285	0,005581	0,048809	0,030817	0,030817	0,084607
s__Dialister_invisust__GCF_000160055	-0,10169	-0,1488	0,154297	-0,05392	0,027928	-0,06785	0,190602	0,205225	0,205225	0,083615
s__Streptococcus_thermophilust__Streptococcus_thermophilus_unclassified	-0,06405	0,133843	-0,01937	-0,05768	-0,08882	0,006873	0,00787	0,078113	0,078113	0,082657
s__Streptococcus_parasanguinist__Streptococcus_parasanguinis_unclassified	-0,0	0,05013	-0,1	0,157607	-0,10337	0,018776	-0,1	-0,10797	-0,1	0,08254

	9255		6499				4567		0797	
s__Flavonifractor_plautiit__GCF_000239295	-0,13386	-0,02791	-0,07985	0,085777	0,035749	0,002293	0,113536	0,044923	0,044923	0,081782
s__Bifidobacterium_animalist__Bifidobacterium_animalis_unclassified	-0,06128	0,069053	0,099241	-0,03024	0,083336	-0,00901	0,034116	0,034665	0,034665	0,079373
s__Haemophilus_sputorumt__Haemophilus_sputorum_unclassified	-0,03604	0,018146	0,150431	-0,0268	0,009068	0,116108	-0,04745	-0,08527	-0,08527	0,07629
s__Bifidobacterium_bifidumt__Bifidobacterium_bifidum_unclassified	-0,11613	0,018537	-0,11554	0,175523	0,049624	-0,01878	0,032792	0,140925	0,140925	0,076265
s__Paraprevotella_clarat__GCF_000233955	0,054682	0,121052	-0,06462	-0,0186	0,031223	0,104653	0,001458	0,050118	0,050118	0,075451
s__Lachnospiraceae_bacterium_1_4_56FAAt__GCF_000218385	-0,06265	0,077971	-0,00658	-0,03024	0,083439	0,03081	0,114505	0,089611	0,089611	0,074386
s__Actinomyces_odontolyticust__Actinomyces_odontolyticus_unclassified	-0,04351	-0,05138	-0,03187	-0,00917	-0,07248	0,065616	-0,0614	-0,07125	-0,07125	0,074201
s__Rothia_mucilaginosat__Rothia_mucilaginosa_unclassified	-0,04351	-0,05138	-0,03187	-0,00917	-0,07248	0,065616	-0,0614	-0,07125	-0,07125	0,074201
s__Solobacterium_mooreit__GCF_000186945	-0,04351	-0,05138	-0,03187	-0,00917	-0,07248	0,065616	-0,0614	-0,07125	-0,07125	0,074201
s__Clostridium_ramosumt__GCF_000154485	0,055179	0,078063	0,042174	0,10402	0,024254	0,131486	-0,17609	-0,16795	-0,16795	0,074094
s__Alistipes_senegalensist__GCF_000312145	-0,09909	-0,00468	-0,07258	-0,02089	-0,16508	-0,15015	0,237575	0,224996	0,224996	0,072738
s__Haemophilus_parahaemolyticust__GCF_000262265	-0,04351	0,199343	0,281027	-0,00917	-0,07248	0,145634	-0,0614	-0,07125	-0,07125	0,071172

s__Enterococcus_gallinarumt__GCF_000157255	-0,07605	-0,08981	-0,05571	-0,01604	-0,01514	0,012326	-0,10733	-0,12455	-0,12455	0,070922
s__Erysipelotrichaceae_bacterium_2_2_44At__GCF_000225685	-0,00411	0,081771	0,047344	-0,03497	0,014492	0,170438	-0,07495	-0,11694	-0,11694	0,070443
s__Parabacteroides_distasonist__Parabacteroides_distasonis_unclassified	-0,07049	0,038123	-0,22721	0,079569	0,163254	-0,00292	0,203226	0,17502	0,17502	0,069163
s__Parabacteroides_merdaet__Parabacteroides_merdae_unclassified	0,052364	0,126029	-0,06686	0,191325	0,023241	0,031436	0,099285	0,142385	0,142385	0,068301
s__Bacteroides_fragilist__GCF_000297735	-0,04351	-0,05138	-0,03187	-0,00917	-0,07248	-0,09122	0,184211	0,172047	0,172047	0,068144
s__Clostridiaceae_bacterium_JC118t__GCF_000313565	-0,04351	-0,05138	-0,03187	-0,00917	-0,07248	-0,09122	0,184211	0,172047	0,172047	0,068144
s__Lachnospiraceae_bacterium_3_1_57FAA_CT1t__GCF_000218405	0,001716	-0,16937	-0,10506	-0,03024	-0,14378	-0,03928	-0,02665	-0,00209	-0,00209	0,068055
s__Holdemania_filiformist__GCF_000157995	-0,13543	-0,00717	-0,0992	0,307791	-0,03963	-0,21053	0,128888	0,14265	0,14265	0,066821
s__Lactobacillus_acidophilust__Lactobacillus_acidophilus_unclassified	-0,01875	0,060787	0,027937	-0,02495	0,021091	0,017535	0,011633	0,026899	0,026899	0,065566
s__Peptostreptococcus_anaerobiust__Peptostreptococcus_anaerobius_unclassified	0,141718	0,065479	-0,04528	-0,01303	-0,10298	-0,1296	-0,08724	-0,10123	-0,10123	0,065225
s__Leuconostoc_lactist__Leuconostoc_lactis_unclassified	-0,06181	0,053695	-0,04528	-0,01303	-0,10298	-0,1296	0,17697	0,17507	0,17507	0,063152
s__Lactococcus_garvieaet__Lactococcus_garvieae_unclassified	-0,06181	0,053695	-0,04528	-0,01303	-0,10298	-0,1296	0,17697	0,17507	0,17507	0,063152
s__Enterococcus_duranst__Enterococcus_durans_unclassified	-0,0	0,013171	-0,0	-0,0	-0,1267	-0,0	-0,0	0,152677	0,152677	0,062869

	7605		5571	1604		9869	0542			
s__Streptococcus_vestibularist__Streptococcus_vestibularis_unclassified	-0,06181	-0,07299	-0,04528	-0,01303	-0,10298	-0,1296	0,051476	0,106479	0,106479	0,061607
s__Erysipelotrichaceae_bacterium_6_1_45t__GCF_000242175	-0,0627	0,282	-0,0065	-0,03187	0,063239	0,196321	-0,09448	-0,13751	-0,13751	0,058889
s__Bacteroides_xylanisolvenst__Bacteroides_xylanisolvens_unclassified	-0,14176	-0,00294	-0,04422	-0,05392	0,010948	0,098419	0,320945	0,265955	0,265955	0,05543
s__Streptococcus_salivariust__Streptococcus_salivarius_unclassified	-0,00259	0,040143	-0,17078	0,082121	0,018387	0,007125	-0,09605	-0,09628	-0,09628	0,051848
s__Bacteroides_doreit__Bacteroides_dorei_unclassified	-0,1495	0,111802	-0,15452	0,111429	0,108457	-0,06709	0,13539	0,132059	0,132059	0,05155
s__Lactobacillus_fermentumt__Lactobacillus_fermentum_unclassified	-0,06181	0,054305	-0,04528	-0,01303	0,03681	0,09973	-0,08724	-0,10123	-0,10123	0,048503
s__Erysipelotrichaceae_bacterium_3_1_53t__GCF_000165065	-0,06181	-0,07299	-0,04528	-0,01303	-0,10298	-0,1296	0,077791	0,142592	0,142592	0,04645
s__Enterococcus_faeciumt__Enterococcus_faecium_unclassified	0,002522	-0,03504	0,069654	-0,02089	-0,09746	-0,06838	0,11146	0,13916	0,13916	0,039968
s__Megasphaera_micronuciformist__GCF_000165735	-0,04495	0,069985	-0,05053	-0,03936	-0,03125	0,106348	-0,16779	-0,21321	-0,21321	0,038319
s__Veillonella_atypicat__Veillonella_atypica_unclassified	0,236132	0,053245	-0,08393	0,069838	0,126061	0,238352	-0,32844	-0,35778	-0,35778	0,032151
s__Prevotella_coprit__GCF_000157935	-0,0473	-0,08466	-0,0992	-0,02856	0,090608	0,050692	0,064438	0,17408	0,17408	0,028073
s__Adlercreutzia_equolifacienst__GCF_000478885	-0,14342	0,243632	-0,10506	-0,03024	0,023326	-0,00069	0,172834	0,147692	0,147692	0,02506

s__Lactobacillus_orist__Lactobacillus_oris_unclassified	0,13805	0,18967	-0,04528	-0,01303	-0,10298	-0,1296	0,038138	0,025834	0,025834	0,02478
s__Coprococcus_eutactust__GCF_000154425	-0,06181	0,10378	-0,04528	-0,01303	-0,10298	-0,1296	0,140009	0,138193	0,138193	0,024516
s__Streptococcus_australist__Streptococcus_australis_unclassified	0,063848	0,195295	0,131312	-0,0268	0,052669	0,186891	-0,11218	-0,13386	-0,13386	0,024088
s__Bacteroides_nordiit__GCF_000273175	-0,06181	0,07745	-0,04528	-0,01303	0,16661	0,153925	0,027468	0,005903	0,005903	0,022824
s__Bifidobacterium_adolescentist__Bifidobacterium_adolescentis_unclassified	-0,128	-0,13788	-0,0548	0,181683	-0,07927	-0,23716	-0,00206	0,065666	0,065666	0,022037
s__Ruminococcus_sp_5_1_39BFAAt__GCF_000159975	-0,09909	-0,0217	-0,07258	0,422064	0,023482	-0,07047	-0,02335	0,009197	0,009197	0,021959
s__Clostridium_citroniaet__GCF_000233455	0,007997	-0,14555	-0,05776	-0,03792	-0,07226	-0,13187	0,162312	0,115335	0,115335	0,02142
s__Granulicatella_eleganst__GCF_000162475	-0,06181	0,271207	-0,04528	-0,01303	0,23049	0,218455	-0,08724	-0,10123	-0,10123	0,020418
s__Bacteroides_cellulosilyticust__Bacteroides_cellulosilyticus_unclassified	-0,03134	-0,06046	-0,04813	-0,03647	-0,02521	-0,19041	0,19455	0,226064	0,226064	0,020363
s__Gordonibacter_pamelaeaet__GCF_000210055	-0,07605	0,063027	-0,05571	-0,01604	0,006582	-0,02762	0,118015	0,083969	0,083969	0,018689
s__Streptococcus_perorist__GCF_000187585	-0,08822	-0,10418	-0,06462	-0,0186	-0,14697	-0,05329	-0,12451	-0,07464	-0,07464	0,017969
s__Streptococcus_macedonicust__GCF_000283635	-0,04351	0,158241	-0,03187	-0,00917	-0,07248	0,148835	0,109782	0,071252	0,071252	0,016657
s__Coprobacter_fastidiosust__GCF_000473955	-0,0	-0,1	-0,0	-0,0	-0,08404	-0,0	0,150509	0,182769	0,182769	0,015877

	9909	1701	7258	2089		9695				
s__Blautia_productat__GCF_000373885	0,040626	0,118779	0,092471	-0,0186	-0,06273	0,189614	0,042737	0,022072	0,022072	0,015499
s__Alistipes_finegoldiit__GCF_000265365	-0,22547	0,106962	0,022778	-0,06017	0,051602	-0,02696	0,208598	0,228348	0,228348	0,014848
s__Lactococcus_phage_P680t__PRJNA213080	0,11509	0,059994	0,067302	-0,02089	-0,06487	0,042585	-0,04498	-0,06279	-0,06279	0,014786
s__Bacteroides_barnesiaet__GCF_000374585	-0,06181	0,08021	-0,04528	-0,01303	0,039284	0,051653	0,069932	0,065135	0,065135	0,013808
s__Megasphaera_elsdeniit__GCF_000283495	-0,04351	-0,05138	-0,03187	-0,00917	-0,07248	0,011203	-0,0614	0,067776	0,067776	0,013629
s__Sutterella_wadsworthensist__GCF_000186505	-0,04351	-0,05138	-0,03187	-0,00917	-0,07248	0,011203	-0,0614	0,067776	0,067776	0,013629
s__Bifidobacterium_catenulatumt__GCF_000173455	-0,11756	0,110758	-0,05049	0,209076	0,000925	0,017764	-0,12574	-0,0237	-0,0237	0,013612
s__Varibaculum_cambrienset__GCF_000420065	-0,06181	0,097701	-0,04528	-0,01303	0,027003	0,041628	-0,08724	-0,10123	-0,10123	0,009642
s__Clostridium_innocuumt__GCF_000371425	0,032088	-0,03408	0,089795	-0,03024	-0,00983	0,178273	-0,09483	-0,12714	-0,12714	0,009458
s__Human_adenovirus_Bt__Human_adenovirus_B_unclassified	-0,04351	-0,05138	-0,03187	-0,00917	-0,07248	0,030407	-0,0614	-0,07125	-0,07125	0,007572
s__Bacteroides_caccaet__Bacteroides_caccae_unclassified	-0,02288	0,050334	-0,07509	0,126485	0,174046	-0,05048	0,013282	0,102172	0,102172	0,006564
s__Alistipes_sp_AP11t__GCF_000321205	-0,04351	0,174682	-0,03187	-0,00917	0,098369	0,100824	-0,0614	-0,07125	-0,07125	0,004543

s__Streptococcus_sanguinist__Streptococcus_sanguinis_unclassified	-0,06181	0,086129	-0,04528	-0,01303	0,017285	0,072116	-0,08724	-0,10123	-0,10123	0,004283
s__Enterococcus_casseliflavust__Enterococcus_casseliflavus_unclassified	-0,10905	-0,05159	-0,07988	-0,02299	-0,18167	-0,16134	-0,07752	-0,10117	-0,10117	0,00165
s__Veillonella_dispart__GCF_000160015	0,110323	-0,05788	0,117768	0,122212	-0,07133	0,001819	-0,34693	-0,38927	-0,38927	0,001199
s__Lactobacillus_rhamnosust__Lactobacillus_rhamnosus_unclassified	0,009368	0,058582	0,110947	0,203803	0,035443	-0,06734	-0,11645	-0,13197	-0,13197	0,000712
s__Bifidobacterium_pseudocatenulatumt__GCF_000173435	-0,13607	0,056978	-0,00759	0,186267	0,195626	0,028134	0,071555	0,099193	0,099193	-0,00066
s__Bacteroides_massiliensist__Bacteroides_massiliensis_unclassified	-0,09909	0,228727	-0,07258	-0,02089	0,148184	0,19299	0,059529	0,031822	0,031822	-0,00686
s__Aggregatibacter_segnist__GCF_000185305	-0,04351	-0,05138	-0,03187	-0,00917	-0,07248	-0,09122	-0,0614	-0,07125	-0,07125	-0,00757
s__Escherichia_hermanniit__GCF_000248015	-0,06181	-0,07299	-0,04528	-0,01303	0,051655	-0,1296	-0,08724	-0,10123	-0,10123	-0,00763
s__Clostridiales_bacterium_1_7_47FAAt__GCF_000155435	-0,06087	0,169229	0,078582	-0,05009	0,129815	0,149217	0,017834	-0,05123	-0,05123	-0,02236
s__Erysipelotrichaceae_bacterium_5_2_54FAAt__GCF_000163515	-0,06181	0,086129	-0,04528	-0,01303	0,124907	0,10653	-0,08724	-0,10123	-0,10123	-0,02394
s__Butyrivibrio_crossotust__GCF_000156015	-0,04351	-0,05138	-0,03187	-0,00917	0,067305	-0,09122	0,102339	0,109485	0,109485	-0,02574
s__Parabacteroides_johnsoniit__Parabacteroides_johnsonii_unclassified	-0,08822	-0,10418	-0,06462	0,479014	-0,14697	-0,18497	0,096192	0,137316	0,137316	-0,02597
s__Ruminococcus_torquest__Ruminococcus_torques_unclassified	-0,0059	-0,0	0,008354	0,054005	-0,18221	0,002745	0,129111	0,148146	0,148146	-0,0

		2935								3265
s__Lachnospiraceae_bacterium_9_1_43BFAAt__GCF_000209445	-0,00489	0,272278	0,066828	-0,02299	0,167842	0,221892	-0,08304	-0,1196	-0,1196	-0,03339
s__Leuconostoc_mesenteroidest__Leuconostoc_mesenteroides_unclassified	-0,04351	-0,05138	-0,03187	-0,00917	-0,07248	-0,09122	-0,0614	0,053873	0,053873	-0,03483
s__Clostridium_asparagiformet__GCF_000158075	-0,11639	0,013505	0,034486	0,21819	0,046374	0,025742	-0,07973	-0,09182	-0,09182	-0,03592
s__Lactococcus_phage_bIL67t__PRJNA32321	-0,04351	-0,05138	-0,03187	-0,00917	0,07766	0,049612	-0,0614	-0,07125	-0,07125	-0,03786
s__Streptococcus_mitis_oralis_pneumoniaet__Streptococcus_mitis_oralis_pneumoniae_unclassified	0,00867	0,095014	-0,1464	-0,04214	0,037496	0,118197	-0,28207	-0,26292	-0,26292	-0,03824
s__Proteus_mirabilist__Proteus_mirabilis_unclassified	-0,06181	-0,07299	-0,04528	-0,01303	-0,10298	-0,0493	-0,08724	-0,10123	-0,10123	-0,03844
s__Lactobacillus_casei_paracaseit__Lactobacillus_casei_paracasei_unclassified	-0,06472	0,01672	0,00707	-0,03187	-0,08044	-0,19807	-0,15304	-0,0964	-0,0964	-0,03889
s__Klebsiella_oxytocat__Klebsiella_oxytoca_unclassified	0,108617	-0,04022	0,119003	0,275219	-0,0239	0,03247	-0,14958	-0,15197	-0,15197	-0,04029
s__Actinomyces_graevenitziit__Actinomyces_graevenitzii_unclassified	-0,06181	-0,07299	-0,04528	-0,01303	-0,10298	-0,01718	-0,08724	-0,10123	-0,10123	-0,04061
s__Atopobium_minutumt__GCF_000364325	-0,04351	-0,05138	-0,03187	-0,00917	0,12253	0,046411	-0,0614	-0,07125	-0,07125	-0,04089
s__Pediococcus_acidilacticit__Pediococcus_acidilactici_unclassified	-0,04351	-0,05138	-0,03187	-0,00917	0,12253	0,046411	-0,0614	-0,07125	-0,07125	-0,04089
s__Klebsiella_pneumoniaet__Klebsiella_pneumoniae_unclassified	0,160251	-0,03222	0,11853	-0,03792	-0,00583	-0,0228	-0,14298	-0,12948	-0,12948	-0,04188

s__Citrobacter_freundiit__Citrobacter_freundii_unclassified	0,023869	-0,10418	0,092284	-0,0186	0,025577	-0,0385	-0,12451	-0,14448	-0,14448	-0,04204
s__Anaerotruncus_colihominist__GCF_000154565	-0,00575	0,013641	0,05516	-0,03497	0,03501	-0,02149	0,154334	0,111862	0,111862	-0,04334
s__Lachnospiraceae_bacterium_2_1_58FAAt__GCF_000218465	0,122494	-0,01894	-0,00198	-0,05392	0,036055	-0,02927	-0,02214	-0,05819	-0,05819	-0,04377
s__Enterobacter_cloacaet__Enterobacter_cloacae_unclassified	0,144188	-0,04674	0,082825	-0,03187	0,142093	-0,03144	-0,15668	-0,18664	-0,18664	-0,04472
s__Lactobacillus_phage_Lc_Nut__Lactobacillus_phage_Lc_Nu_unclassified	0,09576	0,103488	0,174967	-0,01303	0,014544	0,197724	-0,08724	-0,10123	-0,10123	-0,04542
s__Lachnospiraceae_bacterium_5_1_57FAAt__GCF_000218425	-0,07605	0,037163	-0,05571	-0,01604	-0,05329	0,045216	0,101102	0,176426	0,176426	-0,05219
s__Eubacterium_sp_3_1_31t__Eubacterium_sp_3_1_31_unclassified	-0,07605	0,039514	-0,05571	-0,01604	0,060113	0,086177	-0,10733	-0,04557	-0,04557	-0,05251
s__Phascolarctobacterium_succinatutenst__GCF_000188175	0,282009	0,184578	-0,07258	-0,02089	0,082153	0,093506	-0,04498	-0,06875	-0,06875	-0,05301
s__Bacteroides_phage_B124_14t__PRJNA82753	-0,04351	-0,05138	-0,03187	-0,00917	-0,07248	-0,0016	-0,0614	0,0643	0,0643	-0,05603
s__Lactococcus_phage_BM13t__PRJNA213076	-0,04351	0,154131	-0,03187	-0,00917	0,177755	0,113627	-0,0614	-0,07125	-0,07125	-0,05906
s__Lactobacillus_plantarumt__Lactobacillus_plantarum_unclassified	-0,04351	0,12536	-0,03187	-0,00917	-0,07248	-0,09122	0,113504	0,106009	0,106009	-0,06209
s__Lactobacillus_salivariust__Lactobacillus_salivarius_unclassified	-0,04351	0,12536	-0,03187	-0,00917	-0,07248	-0,09122	0,113504	0,106009	0,106009	-0,06209
s__Clostridium_sporogenest__Clostridium_sporogenes_unclassified	-0,0	0,12536	-0,0	-0,0	-0,07248	-0,0	0,113504	0,106009	0,106009	-0,0

	4351		3187	0917		9122				6209
s__Anaerococcus_hydrogenalist__Anaerococcus_hydrogenalis_unclassified	-0,04351	0,12536	-0,03187	-0,00917	-0,07248	-0,09122	0,113504	0,106009	0,106009	-0,06209
s__Anaerococcus_obesiensist__GCF_000311745	-0,04351	0,12536	-0,03187	-0,00917	-0,07248	-0,09122	0,113504	0,106009	0,106009	-0,06209
s__Anaerococcus_prevotiit__Anaerococcus_prevotii_unclassified	-0,04351	0,12536	-0,03187	-0,00917	-0,07248	-0,09122	0,113504	0,106009	0,106009	-0,06209
s__Anaerococcus_vaginalist__GCF_000163295	-0,04351	0,12536	-0,03187	-0,00917	-0,07248	-0,09122	0,113504	0,106009	0,106009	-0,06209
s__Finegoldia_magnat__Finegoldia_magna_unclassified	-0,04351	0,12536	-0,03187	-0,00917	-0,07248	-0,09122	0,113504	0,106009	0,106009	-0,06209
s__Haemophilus_influenzaet__Haemophilus_influenzae_unclassified	0,125156	-0,07299	-0,04528	-0,01303	0,009997	-0,02821	-0,08724	-0,10123	-0,10123	-0,06232
s__Lactococcus_raffinolactist__GCF_000327305	-0,06181	-0,07299	-0,04528	-0,01303	0,107856	0,059011	-0,08724	-0,10123	-0,10123	-0,06247
s__Clostridium_leptumt__GCF_000154345	-0,13543	-0,07453	-0,0992	-0,02856	-0,01198	-0,15826	0,041621	0,005584	0,005584	-0,06442
s__Bacteroides_coprocolat__GCF_000154845	-0,07605	0,036989	-0,05571	-0,01604	0,194836	0,192411	0,116241	0,112285	0,112285	-0,06463
s__Human_adenovirus_Ft__PRJNA14487	-0,04351	0,215784	-0,03187	-0,00917	0,160497	0,139233	-0,0614	-0,07125	-0,07125	-0,06814
s__Bacteroides_intestinalist__GCF_000172175	-0,08822	-0,00746	0,264408	-0,0186	-0,06288	-0,08182	-0,12451	-0,08278	-0,08278	-0,06852
s__Clostridium_clostridioformet__GCF_000371525	-0,04351	0,191123	-0,03187	-0,00917	0,112175	0,152036	-0,0614	-0,07125	-0,07125	-0,0742

s__Subdoligranulum_variabilet__GCF_000157955	-0,04351	0,191123	-0,03187	-0,00917	0,112175	0,152036	-0,0614	-0,07125	-0,07125	-0,0742
s__Collinsella_intestinalist__GCF_000156175	0,118739	-0,07299	0,174855	-0,01303	-0,10298	-0,047	-0,08724	-0,10123	-0,10123	-0,07535
s__Erysipelotrichaceae_bacterium_21_3t__GCF_000242195	0,063438	0,115971	0,149931	-0,0268	0,069099	0,175172	-0,00704	-0,03205	-0,03205	-0,08243
s__Morganella_morganiit__GCF_000286435	0,171737	-0,05138	0,269438	-0,00917	0,070757	-0,09122	-0,0614	-0,07125	-0,07125	-0,08329
s__Bifidobacterium_dentiumt__Bifidobacterium_dentium_unclassified	-0,0258	-0,07231	0,161218	-0,02495	-0,12412	-0,09109	-0,10028	-0,12617	-0,12617	-0,08616
s__Tannerella_forsythiat__GCF_000238215	0,203794	0,219894	-0,03187	-0,00917	-0,07248	0,104025	-0,0614	-0,07125	-0,07125	-0,08934
s__Streptococcus_infantist__Streptococcus_infantis_unclassified	-0,06181	0,086129	-0,04528	-0,01303	0,017285	0,005581	-0,08724	-0,10123	-0,10123	-0,09232
s__Sutterella_wadsworthensist__Sutterella_wadsworthensis_unclassified	-0,15434	0,007658	-0,08572	0,164936	0,087678	-0,05143	0,094355	0,10712	0,10712	-0,09285
s__Enterococcus_aviumt__Enterococcus_avium_unclassified	-0,13543	-0,08321	0,012298	-0,02856	-0,00829	-0,03117	-0,19114	-0,22179	-0,22179	-0,09422
s__Ruminococcus_gnavust__Ruminococcus_gnavus_unclassified	0,172793	0,143664	-0,10527	-0,12471	0,255864	0,179523	-0,22645	-0,29196	-0,29196	-0,09523
s__Escherichia_colit__Escherichia_coli_unclassified	0,275892	0,045689	0,153959	-0,11226	0,032204	0,186922	-0,39667	-0,41961	-0,41961	-0,09648
s__Faecalibacterium_prausnitziit__GCF_000210735	-0,04351	-0,05138	-0,03187	-0,00917	0,081111	-0,09122	-0,0614	-0,07125	-0,07125	-0,09843
s__Campylobacter_concisust__GCF_000017725	-0,0	-0,0	-0,0	-0,0	-0,09354	0,106588	-0,0	-0,0995	-0,0995	-0,0

	1616	3858	7988	2299			8132			9857
s__Anaerostipes_caccaet__GCF_000154305	-0,07605	0,027758	-0,05571	-0,01604	0,105947	-0,04227	0,007471	0,02351	-0,02351	-0,10102
s__Fusobacterium_nucleatumt__Fusobacterium_nucleatum_unclassified	0,081385	0,02754	-0,05571	-0,01604	-0,1267	-0,05875	-0,10733	-0,12455	-0,12455	-0,10147
s__Enterococcus_faecalist__Enterococcus_faecalis_unclassified	0,043432	-0,08261	0,025774	-0,0268	-0,14376	-0,07612	-0,1126	-0,07837	-0,07837	-0,10647
s__Clostridium_difficilet__Clostridium_difficile_unclassified	0,073327	0,01401	0,034651	-0,02495	0,169183	0,012719	-0,16701	-0,19379	-0,19379	-0,12102
s__Haemophilus_haemolyticust__GCF_000262285	0,099532	-0,02499	0,049642	-0,02299	-0,1128	0,022961	-0,1539	-0,17859	-0,17859	-0,12189
s__Lactobacillus_zeaet__GCF_000260435	0,194635	-0,05138	-0,03187	-0,00917	-0,07248	0,078419	-0,0614	-0,07125	-0,07125	-0,13326
s__Streptococcus_pseudopneumoniaet__Streptococcus_pseudopneumoniae_unclassified	0,180896	-0,05138	-0,03187	-0,00917	-0,07248	0,12643	-0,0614	-0,07125	-0,07125	-0,13326
s__Actinobaculum_schaaliit__GCF_000411135	-0,04351	-0,05138	0,292616	-0,00917	-0,07248	-0,09122	-0,0614	-0,07125	-0,07125	-0,13326
s__Prevotella_bergensist__GCF_000160535	-0,04351	-0,05138	-0,03187	-0,00917	-0,07248	-0,09122	-0,0614	-0,07125	-0,07125	-0,13326
s__Prevotella_buccalist__GCF_000177075	-0,04351	-0,05138	-0,03187	-0,00917	-0,07248	-0,09122	-0,0614	-0,07125	-0,07125	-0,13326
s__Prevotella_disienst__Prevotella_disiens_unclassified	-0,04351	-0,05138	-0,03187	-0,00917	-0,07248	-0,09122	-0,0614	-0,07125	-0,07125	-0,13326
s__Prevotella_timonensist__GCF_000177055	-0,04351	-0,05138	-0,03187	-0,00917	-0,07248	-0,09122	-0,0614	-0,07125	-0,07125	-0,13326

s__Lachnospiraceae_bacterium_4_1_37FAAt__GCF_000191805	-0,04351	-0,05138	-0,03187	-0,00917	-0,07248	-0,09122	-0,0614	-0,07125	-0,07125	-0,13326
s__Lachnospiraceae_bacterium_6_1_63FAAt__GCF_000209425	-0,04351	-0,05138	-0,03187	-0,00917	-0,07248	-0,09122	-0,0614	-0,07125	-0,07125	-0,13326
s__Campylobacter_curvust__Campylobacter_curvus_unclassified	-0,04351	-0,05138	-0,03187	-0,00917	-0,07248	-0,09122	-0,0614	-0,07125	-0,07125	-0,13326
s__Human_adenovirus_Dt__Human_adenovirus_D_unclassified	-0,04351	-0,05138	-0,03187	-0,00917	-0,07248	-0,09122	-0,0614	-0,07125	-0,07125	-0,13326
s__Bacteroides_sp_3_2_5t__GCF_000159855	-0,06181	0,083236	0,162395	-0,01303	-0,10298	0,025857	0,038499	0,035777	0,035777	-0,14482
s__Bacteroides_thetaiotaomicront__Bacteroides_thetaiotaomicron_unclassified	-0,04312	0,015482	-0,04453	-0,0651	0,090426	0,01458	0,118257	0,102276	0,102276	-0,14825
s__Bacteroides_eggerthiit__Bacteroides_eggerthii_unclassified	0,045333	0,17081	-0,06462	-0,0186	0,219621	0,146366	-0,12451	-0,14448	-0,14448	-0,15856
s__Veillonella_rattit__GCF_000315505	-0,06181	-0,07299	-0,04528	-0,01303	0,048869	-0,1296	-0,08724	-0,10123	-0,10123	-0,16267
s__Prevotella_biviat__Prevotella_bivia_unclassified	0,145	-0,07299	-0,04528	-0,01303	0,083818	0,037887	-0,08724	-0,10123	-0,10123	-0,18932
s__Lactobacillus_gasserit__Lactobacillus_gasseri_unclassified	0,054701	0,015044	-0,05571	-0,01604	0,010531	-0,03264	-0,00357	-0,01939	-0,01939	-0,19071
s__Veillonella_parvulat__Veillonella_parvula_unclassified	0,122894	0,032529	0,089387	0,007777	-0,0019	0,082123	-0,27851	-0,32607	-0,32607	-0,21581
s__Bifidobacterium_brevet__Bifidobacterium_breve_unclassified	0,101451	-0,06639	-0,08313	0,14979	0,234865	0,007986	-0,08143	-0,08655	-0,08655	-0,23071
s__Clostridium_perfringenst__Clostridium_perfringens_unclassified	0,001271	-0,1	0,088657	-0,0	-0,07039	-0,1	-0,1	-0,16227	-0,1	-0,2

		1701		2089		1042	3985		6227	8688

Supplementary Table S6. Output from the microbeMASST.

Ala-CDCA	Asn-UDCA	Citrulline-CA	Ser-CDCA	Val-DCA	Ile-Leu-DCA
Clostridium sordelli AO32	Ruminococcus gnavus ATCC29149	Bifidobacterium adolescentis L2 32	Bacillus sp. BK100	Streptomyces sp.	Escherichia coli
Clostridium scindens ATCC35704	Pseudarthrobacter oxydans	Bifidobacterium adolescentis Strain L2-32	Roseateles sp. YR242	Streptomyces sp. BRB-045	Clostridium cadaveris CC88A
	Enterococcus faecium 513	Bifidobacterium angulatum F16 22	Bacillus subtilis	Niallia circulans	Collinsella sp. 4 8 47FAA
		Bifidobacterium breve EX336960VC18	Bacillus tequilensis	Streptomyces sp. BRB-013	Fusobacterium mortiferum
		Bifidobacterium breve EX336960VC19		Streptomyces sp. BRB-047	Clostridium sporogenes ATCC 15579
		Bifidobacterium breve HPH0326		Paracoccus homiensis	Bifidobacterium adolescentis Strain L2-32
		Bifidobacterium breve JCP7499		Bacillus subtilis	Clostridium perfringes ATCC13124
		Bifidobacterium breve Strain EX336960VC19			Catabacter hongkongensis AB8 9
		Bifidobacterium longum subsp. longum 35B			Bifidobacterium angulatum F16 22
		Bifidobacterium sp. MSTE12			Enterococcus faecium TX1330
		Clostridiales sp. OBRC5 5			Streptomyces sp.
		Clostridium sp. 7 2 43FAA			Fusobacterium ulcerans, 12 1B
		Clostridium symbiosum WAL 14673			Clostridium sordelli AO32
		Enterococcus faecalis SF19			Clostridium histolytica AO25
		Enterococcus faecium 503			
		Enterococcus faecium 513			
		Enterococcus faecium E417			
		Enterococcus faecium ERV102			

		Enterococcus faecium ERV165			
		Enterococcus faecium ERV99			
		Fusobacterium nucleatum CTI 03			
		Lachnospiraceae sp. CC70A			
		Lachnospiraceae sp. F0167			

Supplementary Table S7. Spearman correlation microbe and MCBAs adjusted p values

Feature	CDCAAla	CDCASer	UDCAAsn	CACys	CACitrulline	CDCATyr	DCAVal	DCAIle	DCALeu	DCAPro
**s__Actinobaculum_schaalii**	0,875137	0,873812	0,081413	0,961352	0,843407	0,842131	0,843407	0,843407	0,843407	0,74096
**s__Actinobaculum_unclassified**	0,875137	0,873812	0,081413	0,961352	0,843407	0,842131	0,843407	0,843407	0,843407	0,74096
**s__Actinomyces_graevenitzii**	0,843407	0,843407	0,875137	0,961352	0,825491	0,961352	0,843407	0,825491	0,825491	0,890173
**s__Actinomyces_johnsonii**	0,875137	0,873812	0,909092	0,961352	0,843407	0,842131	0,843407	0,875137	0,875137	0,825491
**s__Actinomyces_odontolyticus**	0,875137	0,873812	0,909092	0,961352	0,843407	0,843407	0,843407	0,843407	0,843407	0,843407
**s__Varibaculum_cambriense**	0,843407	0,83439	0,875137	0,961352	0,930575	0,883457	0,843407	0,825491	0,825491	0,961352
**s__Rothia_mucilaginosa**	0,875137	0,873812	0,909092	0,961352	0,843407	0,843407	0,843407	0,843407	0,843407	0,843407
**s__Bifidobacterium_adolescentis**	0,753299	0,724253	0,873812	0,515502	0,843407	0,253105	0,991534	0,843407	0,843407	0,94515
**s__Bifidobacterium_angulatum**	0,843407	0,843407	0,875137	0,961352	0,939383	0,875137	0,936286	0,825491	0,825491	0,842131
**s__Bifidobacterium_animalis**	0,844181	0,843407	0,827904	0,914984	0,843407	0,962061	0,909092	0,909092	0,909092	0,843407
**s__Bifidobacterium_bifidum**	0,809515	0,956249	0,811472	0,542576	0,875137	0,956117	0,909092	0,705764	0,705764	0,843407
**s__Bifidobacterium_breve**	0,825491	0,843407	0,843407	0,675728	0,260055	0,96682	0,843407	0,843407	0,843407	0,277255
**s__Bifidobacterium_catenulatum**	0,805428	0,825491	0,875137	0,369026	0,995031	0,959511	0,753299	0,940655	0,940655	0,961352
**s__Bifidobacterium_dentium**	0,934596	0,843407	0,591464	0,937261	0,757612	0,84235	0,827904	0,753299	0,753299	0,843407
**s__Bifidobacterium_longum**	0,940655	0,961352	0,961352	0,925074	0,909092	0,875137	0,909092	0,873812	0,873812	0,842131
**s__Bifidobacterium_pseudocatenulatum**	0,73259	0,871984	0,967824	0,482376	0,45288	0,92671	0,843407	0,827904	0,827904	0,995286
**s__Adlercreutzia_equolifaciens**	0,70144	0,236937	0,825491	0,914984	0,940914	0,995286	0,54667	0,688284	0,688284	0,937261
**s__Atopobium_minutum**	0,875137	0,873812	0,909092	0,961352	0,771391	0,875137	0,843407	0,843407	0,843407	0,889546
**s__Collinsella_aerofaciens**	0,965133	0,843407	0,843407	0,524816	0,722192	0,842131	0,268946	0,080846	0,080846	0,438089
**s__Collinsella_intestinalis**	0,800687	0,843407	0,542576	0,961352	0,825491	0,875137	0,843407	0,825491	0,825491	0,843407
**s__Collinsella_tanakaei**	0,875137	0,873812	0,909092	0,961352	0,731461	0,842131	0,405928	0,465972	0,465972	0,585515
**s__Collinsella_unclassified**	0,843407	0,843407	0,753299	0,961352	0,753299	0,84235	0,957188	0,977743	0,977743	0,843407
**s__Eggerthella_lenta**	0,829492	0,873933	0,940655	0,800687	0,532912	0,879391	0,842648	0,829492	0,829492	0,253105

**s__Eggerthella_unclassified**	0,54498	0,909092	0,825491	0,873812	0,843407	0,902972	0,961352	0,972163	0,972163	0,059322
**s__Gordonibacter_pamelaeae**	0,843407	0,843407	0,873812	0,961352	0,971338	0,927605	0,801001	0,843407	0,843407	0,956117
**s__Olsenella_unclassified**	0,250694	0,705764	0,909092	0,961352	0,843407	0,842131	0,843407	0,843407	0,843407	0,829492
**s__Bacteroides_barnesiae**	0,843407	0,843407	0,875137	0,961352	0,897976	0,873812	0,843407	0,843407	0,843407	0,961352
**s__Bacteroides_caccae**	0,940914	0,875137	0,843407	0,753299	0,543343	0,875137	0,961352	0,825491	0,825491	0,971338
**s__Bacteroides_cellulosilyticus**	0,911353	0,849815	0,875137	0,909092	0,937261	0,465972	0,458205	0,290409	0,290409	0,950932
**s__Bacteroides_clarus**	0,875137	0,675334	0,842131	0,930575	0,940655	0,70144	0,585515	0,749471	0,749471	0,731461
**s__Bacteroides_coprocola**	0,843407	0,909092	0,873812	0,961352	0,458205	0,465484	0,809515	0,825491	0,825491	0,843407
**s__Bacteroides_coprophilus**	0,875137	0,873812	0,909092	0,961352	0,843407	0,842131	0,585515	0,581164	0,581164	0,825491
**s__Bacteroides_dorei**	0,677094	0,825491	0,650123	0,825491	0,825491	0,843407	0,735241	0,747716	0,747716	0,873812
**s__Bacteroides_eggerthii**	0,875137	0,554324	0,843407	0,956117	0,32546	0,691434	0,753299	0,700991	0,700991	0,611627
**s__Bacteroides_faecis**	0,842131	0,83439	0,843407	0,260055	0,843407	0,961352	0,443203	0,32546	0,32546	0,250948
**s__Bacteroides_finegoldii**	0,92671	0,843407	0,843407	0,948064	0,54667	0,873812	0,585515	0,753299	0,753299	0,465972
**s__Bacteroides_fragilis**	0,133108	0,700489	0,688284	0,961352	0,827904	0,843407	0,07763	0,042747	0,042747	0,753299
**s__Bacteroides_intestinalis**	0,843407	0,968028	0,14688	0,956117	0,843407	0,843407	0,753299	0,843407	0,843407	0,843407
**s__Bacteroides_massiliensis**	0,827904	0,286121	0,843407	0,948064	0,686097	0,463321	0,85495	0,909092	0,909092	0,970989
**s__Bacteroides_nordii**	0,843407	0,843407	0,875137	0,961352	0,570163	0,650123	0,928228	0,972822	0,972822	0,940914
**s__Bacteroides_ovatus**	0,864355	0,853085	0,873812	0,843407	0,873812	0,825491	0,227968	0,340798	0,340798	0,825491
**s__Bacteroides_plebeius**	0,873812	0,843407	0,842131	0,930575	0,918478	0,543343	0,843407	0,909092	0,909092	0,792301
**s__Bacteroides_salyersiae**	0,92697	0,914208	0,843407	0,001144	0,825491	0,691434	0,554324	0,340798	0,340798	0,731461
**s__Bacteroides_sp_3_2_5**	0,843407	0,843407	0,585515	0,961352	0,825491	0,934596	0,902972	0,909092	0,909092	0,700991
**s__Bacteroides_stercoris**	0,939943	0,843407	0,827904	0,925074	0,771391	0,961352	0,689488	0,753299	0,753299	0,842131
**s__Bacteroides_thetaiotaomicron**	0,876923	0,961352	0,875137	0,843407	0,843407	0,961352	0,801001	0,825491	0,825491	0,686097
**s__Bacteroides_uniformis**	0,939943	0,843407	0,842131	0,949005	0,801001	0,722319	0,135074	0,094549	0,094549	0,219392
**s__Bacteroides_vulgatus**	0,92671	0,829492	0,280404	0,884693	0,974522	0,985357	0,842648	0,85413	0,85413	0,842131

**s__Bacteroides_xylanisolvens**	0,705764	0,988372	0,875137	0,873812	0,961352	0,829492	0,041521	0,142634	0,142634	0,873812
**s__Bacteroidales_bacterium_ph8**	0,843407	0,934596	0,825491	0,909092	0,705764	0,74096	0,104407	0,237267	0,237267	0,465173
**s__Barnesiella_intestinihominis**	0,481292	0,875181	0,875137	0,431697	0,873812	0,956117	0,499345	0,250948	0,250948	0,705764
**s__Coprobacter_fastidiosus**	0,827904	0,806978	0,843407	0,948064	0,843407	0,840228	0,675334	0,50848	0,50848	0,961352
**s__Dysgonomonas_unclassified**	0,875137	0,873812	0,909092	0,961352	0,843407	0,843407	0,843407	0,843407	0,843407	0,843407
**s__Odoribacter_splanchnicus**	0,670747	0,940655	0,994028	0,909092	0,873812	0,825491	0,236937	0,192156	0,192156	0,585515
**s__Odoribacter_unclassified**	0,827904	0,965652	0,843407	0,948064	0,705764	0,873812	0,875137	0,927605	0,927605	0,873812
**s__Parabacteroides_distasonis**	0,843407	0,903269	0,290409	0,843407	0,585515	0,988372	0,405928	0,542576	0,542576	0,843407
**s__Parabacteroides_goldsteinii**	0,843407	0,825491	0,843407	0,000111	0,873812	0,825491	0,582098	0,383005	0,383005	0,596549
**s__Parabacteroides_johnsonii**	0,843407	0,825491	0,843407	0,000111	0,688284	0,496441	0,842131	0,725399	0,725399	0,934596
**s__Parabacteroides_merdae**	0,873812	0,753299	0,843407	0,465972	0,940914	0,910793	0,827904	0,705764	0,705764	0,843407
**s__Parabacteroides_unclassified**	0,971338	0,952392	0,554324	0,843407	0,776294	0,875137	0,843407	0,95758	0,95758	0,956117
**s__Porphyromonas_somerae**	0,875137	0,873812	0,909092	0,961352	0,843407	0,842131	0,715446	0,753299	0,753299	0,747716
**s__Tannerella_forsythia**	0,402864	0,32546	0,909092	0,961352	0,843407	0,825491	0,843407	0,843407	0,843407	0,843407
**s__Alloprevotella_tannerae**	0,875137	0,873812	0,909092	0,961352	0,843407	0,842131	0,843407	0,843407	0,843407	0,675334
**s__Alloprevotella_unclassified**	0,875137	0,873812	0,909092	0,961352	0,843407	0,842131	0,843407	0,843407	0,843407	0,675334
**s__Paraprevotella_clara**	0,873812	0,78235	0,843407	0,956117	0,911621	0,825491	0,99296	0,875137	0,875137	0,843407
**s__Paraprevotella_unclassified**	0,961352	0,843407	0,843407	0,940914	0,919992	0,862652	0,875137	0,843407	0,843407	0,873812
**s__Paraprevotella_xylaniphila**	0,722319	0,842131	0,875137	0,961352	0,961352	0,972822	0,843407	0,99275	0,99275	0,701084
**s__Prevotella_bergensis**	0,875137	0,873812	0,909092	0,961352	0,843407	0,842131	0,843407	0,843407	0,843407	0,74096
**s__Prevotella_bivia**	0,700489	0,843407	0,875137	0,961352	0,843407	0,903269	0,843407	0,825491	0,825491	0,465972
**s__Prevotella_buccae**	0,843407	0,843407	0,688284	0,961352	0,961352	0,862652	0,94515	0,875137	0,875137	0,290409
**s__Prevotella_buccalis**	0,875137	0,873812	0,909092	0,961352	0,843407	0,842131	0,843407	0,843407	0,843407	0,74096
**s__Prevotella_copri**	0,875137	0,843407	0,827904	0,925074	0,843407	0,875137	0,843407	0,543343	0,543343	0,926886
**s__Prevotella_disiens**	0,875137	0,873812	0,909092	0,961352	0,843407	0,842131	0,843407	0,843407	0,843407	0,74096

**s__Prevotella_oralis**	0,431697	0,873812	0,055492	0,961352	0,811228	0,843407	0,843407	0,843407	0,843407	0,771391
**s__Prevotella_stercorea**	0,875137	0,873812	0,909092	0,961352	0,843407	0,842131	0,843407	0,875137	0,875137	0,825491
**s__Prevotella_timonensis**	0,875137	0,873812	0,909092	0,961352	0,843407	0,842131	0,843407	0,843407	0,843407	0,74096
**s__Alistipes_finegoldii**	0,292917	0,825491	0,940914	0,851924	0,873812	0,930575	0,372119	0,286121	0,286121	0,961352
**s__Alistipes_indistinctus**	0,801001	0,909092	0,843407	0,937261	0,873812	0,843407	0,753299	0,725399	0,725399	0,843407
**s__Alistipes_onderdonkii**	0,148457	0,843407	0,982523	0,843407	0,956249	0,843407	0,096186	0,045392	0,045392	0,842131
**s__Alistipes_putredinis**	0,478984	0,843407	0,954291	0,74096	0,843407	0,731461	0,290409	0,127995	0,127995	0,691434
**s__Alistipes_senegalensis**	0,827904	0,977743	0,843407	0,948064	0,581164	0,675334	0,253105	0,293481	0,293481	0,843407
**s__Alistipes_shahii**	0,54667	0,961352	0,843407	0,318185	0,843407	0,939383	0,753299	0,443203	0,443203	0,827904
**s__Alistipes_sp_AP11**	0,875137	0,542576	0,909092	0,961352	0,829492	0,826843	0,843407	0,843407	0,843407	0,977966
**s__Alistipes_sp_HGB5**	0,875137	0,873812	0,909092	0,961352	0,843407	0,842131	0,843407	0,843407	0,843407	0,70144
**s__Alistipes_unclassified**	0,961352	0,340798	0,777866	0,909092	0,215398	0,919066	0,83439	0,843407	0,843407	0,843407
**s__Gemella_haemolysans**	0,290409	0,675334	0,909092	0,961352	0,843407	0,843407	0,843407	0,843407	0,843407	0,842131
**s__Gemella_unclassified**	0,747716	0,277255	0,875137	0,961352	0,961144	0,749471	0,843407	0,825491	0,825491	0,843407
**s__Facklamia_unclassified**	0,875137	0,753299	0,909092	0,961352	0,843407	0,842131	0,82317	0,825491	0,825491	0,843407
**s__Granulicatella_elegans**	0,843407	0,127995	0,875137	0,961352	0,277255	0,33261	0,843407	0,825491	0,825491	0,950932
**s__Granulicatella_unclassified**	0,962133	0,537423	0,843407	0,940914	0,842131	0,825491	0,650123	0,532912	0,532912	0,843407
**s__Enterococcus_avium**	0,735241	0,843407	0,961352	0,925074	0,965652	0,911748	0,465972	0,316398	0,316398	0,842131
**s__Enterococcus_casseliflavus**	0,825491	0,873812	0,843407	0,940914	0,515502	0,591299	0,843407	0,825491	0,825491	0,99275
**s__Enterococcus_durans**	0,843407	0,961352	0,873812	0,961352	0,753299	0,829492	0,973811	0,6574	0,6574	0,843407
**s__Enterococcus_faecalis**	0,875181	0,843407	0,934596	0,930575	0,70144	0,843407	0,825491	0,843407	0,843407	0,825491
**s__Enterococcus_faecium**	0,990219	0,909092	0,843407	0,948064	0,835852	0,843407	0,825491	0,717285	0,717285	0,894918
**s__Enterococcus_gallinarum**	0,843407	0,843407	0,873812	0,961352	0,961352	0,961352	0,825491	0,753299	0,753299	0,843407
**s__Lactobacillus_acidophilus**	0,956117	0,847114	0,92697	0,937261	0,948064	0,960822	0,961352	0,930575	0,930575	0,843407
**s__Lactobacillus_casei_paracasei**	0,843407	0,961352	0,97017	0,909092	0,843407	0,439277	0,656456	0,842131	0,842131	0,901448

**s__Lactobacillus_delbrueckii**	0,875137	0,873812	0,909092	0,961352	0,843407	0,842131	0,843407	0,875137	0,875137	0,825491
**s__Lactobacillus_fermentum**	0,843407	0,873812	0,875137	0,961352	0,909092	0,827904	0,843407	0,825491	0,825491	0,875137
**s__Lactobacillus_gasseri**	0,873812	0,961352	0,873812	0,961352	0,961352	0,909092	0,984711	0,956117	0,956117	0,465972
**s__Lactobacillus_oris**	0,723112	0,465972	0,875137	0,961352	0,825491	0,749471	0,903269	0,934596	0,934596	0,93849
**s__Lactobacillus_plantarum**	0,875137	0,753299	0,909092	0,961352	0,843407	0,842131	0,82317	0,825491	0,825491	0,843407
**s__Lactobacillus_rhamnosus**	0,961352	0,860858	0,825491	0,402864	0,909092	0,843407	0,809515	0,747716	0,747716	0,995286
**s__Lactobacillus_ruminis**	0,843407	0,749471	0,873812	0,961352	0,753299	0,825491	0,825491	0,881819	0,881819	0,753299
**s__Lactobacillus_salivarius**	0,875137	0,753299	0,909092	0,961352	0,843407	0,842131	0,82317	0,825491	0,825491	0,843407
**s__Lactobacillus_zeae**	0,458205	0,873812	0,909092	0,961352	0,843407	0,843407	0,843407	0,843407	0,843407	0,74096
**s__Pediococcus_acidilactici**	0,875137	0,873812	0,909092	0,961352	0,771391	0,875137	0,843407	0,843407	0,843407	0,889546
**s__Leuconostoc_lactis**	0,843407	0,873812	0,875137	0,961352	0,825491	0,749471	0,542093	0,542576	0,542576	0,843407
**s__Leuconostoc_mesenteroides**	0,875137	0,873812	0,909092	0,961352	0,843407	0,842131	0,843407	0,873812	0,873812	0,909092
**s__Leuconostoc_pseudomesenteroides**	0,875137	0,749471	0,909092	0,961352	0,843407	0,842131	0,843407	0,843407	0,843407	0,825491
**s__Weissella_unclassified**	0,875137	0,873812	0,909092	0,961352	0,843407	0,842131	0,733811	0,705764	0,705764	0,656456
**s__Lactococcus_garvieae**	0,843407	0,873812	0,875137	0,961352	0,825491	0,749471	0,542093	0,542576	0,542576	0,843407
**s__Lactococcus_lactis**	0,961352	0,9463	0,913254	0,897976	0,849815	0,995149	0,843407	0,85413	0,85413	0,649303
**s__Lactococcus_raffinolactis**	0,843407	0,843407	0,875137	0,961352	0,825491	0,858632	0,843407	0,825491	0,825491	0,843407
**s__Streptococcus_australis**	0,843407	0,454929	0,74928	0,930575	0,873812	0,481292	0,825491	0,74096	0,74096	0,940655
**s__Streptococcus_infantarius**	0,843407	0,843407	0,875137	0,961352	0,825491	0,880822	0,909092	0,843407	0,843407	0,843407
**s__Streptococcus_infantis**	0,843407	0,843407	0,875137	0,961352	0,961144	0,972822	0,843407	0,825491	0,825491	0,842131
**s__Streptococcus_lutetiensis**	0,70144	0,753299	0,825491	0,914984	0,934596	0,843407	0,688284	0,825491	0,825491	0,753299
**s__Streptococcus_macedonicus**	0,875137	0,6131	0,909092	0,961352	0,843407	0,682329	0,825491	0,843407	0,843407	0,961352
**s__Streptococcus_mitis_oralis_pneumoniae**	0,964226	0,842131	0,691434	0,881819	0,906452	0,801001	0,103464	0,148457	0,148457	0,903269
**s__Streptococcus_parasanguinis**	0,842131	0,875137	0,581164	0,620259	0,825491	0,956117	0,694966	0,825491	0,825491	0,843407
**s__Streptococcus_pasteurianus**	0,843407	0,401844	0,875137	0,961352	0,825491	0,54667	0,909092	0,995031	0,995031	0,843407

**s__Streptococcus_peroris**	0,843407	0,825491	0,843407	0,956117	0,688284	0,873812	0,753299	0,843407	0,843407	0,958409
**s__Streptococcus_pseudopneumoniae**	0,523583	0,873812	0,909092	0,961352	0,843407	0,753299	0,843407	0,843407	0,843407	0,74096
**s__Streptococcus_salivarius**	0,990219	0,893595	0,554324	0,843407	0,956919	0,97017	0,842131	0,842131	0,842131	0,873812
**s__Streptococcus_sanguinis**	0,843407	0,843407	0,875137	0,961352	0,961144	0,843407	0,843407	0,825491	0,825491	0,979788
**s__Streptococcus_thermophilus**	0,843407	0,74096	0,956117	0,865781	0,843407	0,970989	0,967085	0,843407	0,843407	0,843407
**s__Streptococcus_vestibularis**	0,843407	0,843407	0,875137	0,961352	0,825491	0,749471	0,873812	0,825491	0,825491	0,843407
**s__Butyricicoccus_pullicaecorum**	0,890173	0,961352	0,842131	0,956117	0,873812	0,825491	0,875137	0,843407	0,843407	0,809816
**s__Clostridiaceae_bacterium_JC118**	0,875137	0,873812	0,909092	0,961352	0,843407	0,842131	0,50069	0,551668	0,551668	0,843407
**s__Clostridium_asparagiforme**	0,809515	0,961352	0,909092	0,33261	0,875137	0,934596	0,843407	0,842131	0,842131	0,909092
**s__Clostridium_bolteae**	0,940914	0,950932	0,801001	0,873812	0,956117	0,843407	0,829492	0,843407	0,843407	0,542576
**s__Clostridium_citroniae**	0,96682	0,694966	0,865781	0,903269	0,843407	0,747716	0,585515	0,81231	0,81231	0,948064
**s__Clostridium_clostridioforme**	0,33593	0,501999	0,961144	0,858981	0,961352	0,764776	0,873812	0,971548	0,971548	0,843407
**s__Clostridium_hathewayi**	0,843407	0,875137	0,862652	0,843407	0,990219	0,843407	0,753299	0,82317	0,82317	0,250948
**s__Clostridium_leptum**	0,735241	0,843407	0,827904	0,925074	0,961352	0,6131	0,883457	0,972822	0,972822	0,843407
**s__Clostridium_nexile**	0,961352	0,250948	0,634864	0,843407	0,810094	0,598417	0,787483	0,705764	0,705764	0,725399
**s__Clostridium_perfringens**	0,994028	0,806978	0,843407	0,948064	0,843407	0,825491	0,714114	0,585515	0,585515	0,094549
**s__Clostridium_sporogenes**	0,875137	0,753299	0,909092	0,961352	0,843407	0,842131	0,82317	0,825491	0,825491	0,843407
**s__Clostridium_symbiosum**	0,747716	0,542576	0,92671	0,843407	0,927605	0,692069	0,883457	0,910749	0,910749	0,806978
**s__Anaerococcus_hydrogenalis**	0,875137	0,753299	0,909092	0,961352	0,843407	0,842131	0,82317	0,825491	0,825491	0,843407
**s__Anaerococcus_obesiensis**	0,875137	0,753299	0,909092	0,961352	0,843407	0,842131	0,82317	0,825491	0,825491	0,843407
**s__Anaerococcus_prevotii**	0,875137	0,753299	0,909092	0,961352	0,843407	0,842131	0,82317	0,825491	0,825491	0,843407
**s__Anaerococcus_vaginalis**	0,875137	0,753299	0,909092	0,961352	0,843407	0,842131	0,82317	0,825491	0,825491	0,843407
**s__Finegoldia_magna**	0,875137	0,753299	0,909092	0,961352	0,843407	0,842131	0,82317	0,825491	0,825491	0,843407
**s__Clostridiales_bacterium_1_7_47FAA**	0,847114	0,558584	0,843407	0,875137	0,749471	0,679704	0,959259	0,873933	0,873933	0,944151
**s__Flavonifractor_plautii**	0,74096	0,92697	0,843407	0,843407	0,909092	0,990912	0,82317	0,875137	0,875137	0,843407

**s__Pseudoflavonifractor_capillosus**	0,843407	0,843407	0,875137	0,961352	0,961144	0,972822	0,875137	0,913254	0,913254	0,843407
**s__Eubacterium_eligens**	0,585515	0,961352	0,843407	0,843407	0,843407	0,843407	0,001427	0,000942	0,000942	0,340798
**s__Eubacterium_hallii**	0,554324	0,825491	0,961352	0,843407	0,909092	0,562167	0,421108	0,169378	0,169378	0,747716
**s__Eubacterium_ramulus**	0,902972	0,601034	0,827904	0,925074	0,537423	0,33593	0,126648	0,061726	0,061726	0,478984
**s__Eubacterium_rectale**	0,071052	0,656456	0,873812	0,843407	0,843407	0,842131	0,465972	0,236937	0,236937	0,059322
**s__Eubacterium_siraeum**	0,825491	0,843407	0,747716	0,903269	0,585515	0,825491	0,784405	0,585515	0,585515	0,722319
**s__Eubacterium_sp_3_1_31**	0,843407	0,897764	0,873812	0,961352	0,852016	0,843407	0,825491	0,875137	0,875137	0,873812
**s__Eubacterium_ventriosum**	0,843407	0,682329	0,777866	0,909092	0,705764	0,290409	0,280404	0,27455	0,27455	0,825491
**s__Anaerostipes_caccae**	0,843407	0,927605	0,873812	0,961352	0,825491	0,881819	0,968028	0,940744	0,940744	0,825906
**s__Anaerostipes_hadrus**	0,570163	0,843407	0,913254	0,753299	0,843407	0,881423	0,843407	0,651689	0,651689	0,056892
**s__Anaerostipes_unclassified**	0,825491	0,881819	0,753299	0,903269	0,829492	0,873812	0,875137	0,843407	0,843407	0,554324
**s__Blautia_producta**	0,890173	0,800687	0,842131	0,956117	0,843407	0,465972	0,879391	0,94515	0,94515	0,961352
**s__Ruminococcus_gnavus**	0,54667	0,70144	0,825491	0,753299	0,178951	0,532912	0,290409	0,081413	0,081413	0,842131
**s__Ruminococcus_obeum**	0,158196	0,915868	0,990912	0,843407	0,940914	0,714114	0,065266	0,003304	0,003304	0,036241
**s__Ruminococcus_torques**	0,961144	0,876219	0,995132	0,875137	0,422916	0,940914	0,825491	0,601034	0,601034	0,977743
**s__Butyrivibrio_crossotus**	0,875137	0,873812	0,909092	0,961352	0,843407	0,842131	0,825491	0,825491	0,825491	0,934596
**s__Coprococcus_catus**	0,827904	0,806978	0,843407	0,948064	0,843407	0,936203	0,918478	0,875137	0,875137	0,686302
**s__Coprococcus_comes**	0,838256	0,961352	0,909092	0,126648	0,825491	0,585515	0,809816	0,259245	0,259245	0,103304
**s__Coprococcus_eutactus**	0,843407	0,825491	0,875137	0,961352	0,825491	0,749471	0,714114	0,722319	0,722319	0,939383
**s__Dorea_formicigenerans**	0,825491	0,961352	0,543343	0,465972	0,875137	0,842131	0,558584	0,250948	0,250948	0,69458
**s__Dorea_longicatena**	0,829492	0,961352	0,936286	0,873812	0,753299	0,843407	0,705439	0,35625	0,35625	0,290409
**s__Dorea_unclassified**	0,879391	0,842131	0,961352	0,865781	0,913254	0,909092	0,603748	0,402864	0,402864	0,554324
**s__Lachnospiraceae_bacterium_1_1_57FAA**	0,825491	0,843407	0,667738	0,909092	0,843407	0,843407	0,910749	0,987668	0,987668	0,842131
**s__Lachnospiraceae_bacterium_1_4_56FAA**	0,843407	0,843407	0,971338	0,914984	0,843407	0,913254	0,822195	0,843407	0,843407	0,843407
**s__Lachnospiraceae_bacterium_2_1_58FAA**	0,771391	0,956117	0,991827	0,873812	0,909092	0,921105	0,94515	0,862652	0,862652	0,875137

**s__Lachnospiraceae_bacterium_3_1_46FAA**	0,843407	0,558584	0,961352	0,875137	0,961352	0,771391	0,825491	0,843407	0,843407	0,417907
**s__Lachnospiraceae_bacterium_3_1_57FAA_CT1**	0,992617	0,558584	0,825491	0,914984	0,70144	0,897976	0,931545	0,991534	0,991534	0,843407
**s__Lachnospiraceae_bacterium_4_1_37FAA**	0,875137	0,873812	0,909092	0,961352	0,843407	0,842131	0,843407	0,843407	0,843407	0,74096
**s__Lachnospiraceae_bacterium_5_1_57FAA**	0,843407	0,908923	0,873812	0,961352	0,873812	0,875137	0,825594	0,542576	0,542576	0,873812
**s__Lachnospiraceae_bacterium_5_1_63FAA**	0,219392	0,825491	0,542576	0,842131	0,847114	0,961352	0,260055	0,127995	0,127995	0,031353
**s__Lachnospiraceae_bacterium_6_1_63FAA**	0,875137	0,873812	0,909092	0,961352	0,843407	0,842131	0,843407	0,843407	0,843407	0,74096
**s__Lachnospiraceae_bacterium_7_1_58FAA**	0,369026	0,875137	0,961352	0,825491	0,957188	0,909092	0,856928	0,843407	0,843407	0,70144
**s__Lachnospiraceae_bacterium_8_1_57FAA**	0,843407	0,995031	0,992118	0,875137	0,460361	0,843407	0,843407	0,801001	0,801001	0,842131
**s__Lachnospiraceae_bacterium_9_1_43BFAA**	0,977228	0,127995	0,843407	0,940914	0,562167	0,316398	0,843407	0,796337	0,796337	0,909092
**s__Roseburia_hominis**	0,369026	0,956117	0,84275	0,602075	0,956117	0,873812	0,236937	0,045392	0,045392	0,262662
**s__Roseburia_intestinalis**	0,825491	0,961352	0,961352	0,800197	0,799777	0,875137	0,529378	0,54667	0,54667	0,001144
**s__Roseburia_inulinivorans**	0,340798	0,825491	0,875137	0,843407	0,843407	0,889827	0,064619	0,031353	0,031353	0,250948
**s__Roseburia_unclassified**	0,971711	0,909092	0,971338	0,843407	0,640023	0,972822	0,843407	0,875137	0,875137	0,956117
**s__Oscillibacter_unclassified**	0,68376	0,916864	0,873812	0,843407	0,875137	0,842131	0,012236	0,012756	0,012756	0,585515
**s__Clostridium_bartlettii**	0,971548	0,939383	0,843407	0,842131	0,925896	0,909092	0,950932	0,965652	0,965652	0,459907
**s__Clostridium_difficile**	0,843407	0,961352	0,909092	0,937261	0,558584	0,961352	0,569348	0,459907	0,459907	0,78235
**s__Clostridium_hiranonis**	0,875137	0,873812	0,909092	0,961352	0,731461	0,842131	0,405928	0,465972	0,465972	0,585515
**s__Peptostreptococcaceae_noname_unclassified**	0,990712	0,873812	0,956117	0,875137	0,554324	0,873812	0,961352	0,997601	0,997601	0,870091
**s__Peptostreptococcus_anaerobius**	0,705764	0,843407	0,875137	0,961352	0,825491	0,749471	0,843407	0,825491	0,825491	0,843407
**s__Anaerotruncus_colihominis**	0,972822	0,961352	0,873812	0,909092	0,909092	0,948064	0,650123	0,825491	0,825491	0,875545
**s__Anaerotruncus_unclassified**	0,372654	0,873812	0,122109	0,961352	0,843407	0,940655	0,843407	0,843407	0,843407	0,934596
**s__Faecalibacterium_prausnitzii**	0,674897	0,340798	0,585515	0,843407	0,972822	0,843407	0,2145	0,340433	0,340433	0,038962
**s__Ruminococcus_bromii**	0,736085	0,843407	0,875137	0,843407	0,909092	0,843407	0,041072	0,035395	0,035395	0,532912
**s__Ruminococcus_callidus**	0,670747	0,875137	0,948064	0,909092	0,909092	0,873812	0,688284	0,340433	0,340433	0,537423
**s__Ruminococcus_flavefaciens**	0,875137	0,873812	0,909092	0,961352	0,749471	0,909092	0,688284	0,809515	0,809515	0,705764

**s__Ruminococcus_lactaris**	0,827904	0,825491	0,843407	0,875137	0,909092	0,879391	0,463321	0,226621	0,226621	0,33261
**s__Ruminococcus_sp_5_1_39BFAA**	0,827904	0,947106	0,843407	0,001144	0,940744	0,843407	0,940914	0,961352	0,961352	0,945482
**s__Subdoligranulum_unclassified**	0,137167	0,843407	0,585515	0,856217	0,753299	0,843407	0,001103	0,00054	0,00054	0,011991
**s__Subdoligranulum_variabile**	0,875137	0,465972	0,909092	0,961352	0,825491	0,663061	0,843407	0,843407	0,843407	0,843407
**s__Catenibacterium_mitsuokai**	0,875137	0,873812	0,909092	0,961352	0,843407	0,842131	0,585515	0,581164	0,581164	0,825491
**s__Coprobacillus_unclassified**	0,875137	0,825491	0,842131	0,875137	0,842131	0,271258	0,465972	0,478984	0,478984	0,842131
**s__Clostridium_innocuum**	0,909092	0,909092	0,843407	0,914984	0,961352	0,535258	0,842131	0,753299	0,753299	0,961352
**s__Clostridium_ramosum**	0,873812	0,843407	0,881819	0,825491	0,939943	0,747975	0,542576	0,562167	0,562167	0,843407
**s__Erysipelotrichaceae_bacterium_21_3**	0,843407	0,809816	0,675334	0,930575	0,843407	0,542576	0,97017	0,909092	0,909092	0,843407
**s__Erysipelotrichaceae_bacterium_2_2_44A**	0,980847	0,843407	0,875137	0,909092	0,961352	0,554324	0,843407	0,806978	0,806978	0,843407
**s__Erysipelotrichaceae_bacterium_3_1_53**	0,843407	0,843407	0,875137	0,961352	0,825491	0,749471	0,843407	0,705439	0,705439	0,875137
**s__Erysipelotrichaceae_bacterium_5_2_54FAA**	0,843407	0,843407	0,875137	0,961352	0,753299	0,825491	0,843407	0,825491	0,825491	0,940655
**s__Erysipelotrichaceae_bacterium_6_1_45**	0,843407	0,103464	0,971485	0,909092	0,843407	0,446623	0,842131	0,725399	0,725399	0,858981
**s__Eubacterium_biforme**	0,873812	0,939943	0,843407	0,956117	0,843407	0,843407	0,431697	0,465972	0,465972	0,650123
**s__Eubacterium_cylindroides**	0,843407	0,843407	0,873812	1,86E-07	0,753299	0,602448	0,883457	0,558584	0,558584	0,482376
**s__Holdemania_filiformis**	0,735241	0,970137	0,827904	0,058196	0,89757	0,359708	0,753299	0,705439	0,705439	0,843407
**s__Holdemania_unclassified**	0,843407	0,875137	0,809515	0,237267	0,843407	0,851426	0,843407	0,753299	0,753299	0,705764
**s__Solobacterium_moorei**	0,875137	0,873812	0,909092	0,961352	0,843407	0,843407	0,843407	0,843407	0,843407	0,843407
**s__Phascolarctobacterium_succinatutens**	0,103464	0,499345	0,843407	0,948064	0,843407	0,842131	0,875137	0,843407	0,843407	0,873812
**s__Anaeroglobus_geminatus**	0,843407	0,843407	0,875137	0,961352	0,825491	0,747975	0,960822	0,965652	0,965652	0,70144
**s__Dialister_invisus**	0,825491	0,682329	0,650123	0,873812	0,92697	0,843407	0,465972	0,398346	0,398346	0,843407
**s__Megamonas_unclassified**	0,875137	0,873812	0,909092	0,961352	0,731461	0,842131	0,405928	0,465972	0,465972	0,585515
**s__Megasphaera_elsdenii**	0,875137	0,873812	0,909092	0,961352	0,843407	0,961352	0,843407	0,843407	0,843407	0,961352
**s__Megasphaera_micronuciformis**	0,875137	0,843407	0,875137	0,897976	0,911621	0,825491	0,562167	0,340798	0,340798	0,903269
**s__Megasphaera_unclassified**	0,875137	0,873812	0,909092	0,961352	0,843407	0,961352	0,843407	0,843407	0,843407	0,961352

**s__Mitsuokella_unclassified**	0,276257	0,873812	0,909092	0,961352	0,875137	0,645725	0,843407	0,843407	0,843407	0,74096
**s__Veillonella_atypica**	0,259245	0,873812	0,843407	0,843407	0,753299	0,250948	0,035395	0,012756	0,012756	0,909092
**s__Veillonella_dispar**	0,825491	0,865121	0,803501	0,773953	0,843407	0,992118	0,019555	0,00384	0,00384	0,994028
**s__Veillonella_parvula**	0,771391	0,909092	0,843407	0,967517	0,992118	0,843407	0,112812	0,036241	0,036241	0,340433
**s__Veillonella_ratti**	0,843407	0,843407	0,875137	0,961352	0,875137	0,749471	0,843407	0,825491	0,825491	0,585515
**s__Veillonella_unclassified**	0,089949	0,827904	0,842131	0,843407	0,843407	0,146287	0,27455	0,064619	0,064619	0,948064
**s__Fusobacterium_mortiferum**	0,843407	0,843407	0,875137	0,961352	0,873812	0,948064	0,439277	0,439871	0,439871	0,722319
**s__Fusobacterium_nucleatum**	0,843407	0,927944	0,873812	0,961352	0,753299	0,859565	0,825491	0,753299	0,753299	0,825491
**s__Burkholderiales_bacterium_1_1_47**	0,89757	0,843407	0,747716	0,903269	0,939943	0,961352	0,843407	0,842131	0,842131	0,570163
**s__Parasutterella_excrementihominis**	0,873812	0,825491	0,843407	0,889954	0,955597	0,97421	0,903269	0,843407	0,843407	0,558584
**s__Sutterella_wadsworthensis**	0,554324	0,932831	0,825491	0,33261	0,902852	0,825491	0,559729	0,501999	0,501999	0,932831
**s__Neisseria_unclassified**	0,747716	0,843407	0,875137	0,961352	0,825491	0,972822	0,843407	0,825491	0,825491	0,838737
**s__Bilophila_unclassified**	0,532912	0,916864	0,401844	0,558584	0,990219	0,922763	0,019646	0,012002	0,012002	0,501999
**s__Bilophila_wadsworthia**	0,843407	0,971338	0,809515	0,164977	0,843407	0,554324	0,562167	0,340798	0,340798	0,019468
**s__Desulfovibrio_piger**	0,843407	0,825491	0,875137	0,961352	0,825491	0,749471	0,532912	0,511846	0,511846	0,459907
**s__Campylobacter_concisus**	0,961352	0,902972	0,843407	0,940914	0,842131	0,825491	0,843407	0,827904	0,827904	0,829492
**s__Campylobacter_curvus**	0,875137	0,873812	0,909092	0,961352	0,843407	0,842131	0,843407	0,843407	0,843407	0,74096
**s__Campylobacter_gracilis**	0,843407	0,843407	0,875137	0,961352	0,825491	0,956117	0,843407	0,873812	0,873812	0,753299
**s__Citrobacter_freundii**	0,940655	0,825491	0,842131	0,956117	0,935756	0,902972	0,753299	0,700991	0,700991	0,881819
**s__Citrobacter_unclassified**	0,843407	0,714114	0,909092	0,937261	0,859195	0,843407	0,842131	0,753299	0,753299	0,940744
**s__Enterobacter_cloacae**	0,70144	0,875137	0,843407	0,909092	0,705764	0,910793	0,63012	0,481292	0,481292	0,875137
**s__Escherichia_coli**	0,122109	0,875137	0,650123	0,825491	0,909092	0,481292	0,003297	0,001144	0,001144	0,842131
**s__Escherichia_hermannii**	0,843407	0,843407	0,875137	0,961352	0,873812	0,749471	0,843407	0,825491	0,825491	0,967824
**s__Escherichia_unclassified**	0,843407	0,990912	0,581164	0,843407	0,688284	0,73259	0,705764	0,776417	0,776417	0,621611
**s__Klebsiella_oxytoca**	0,825491	0,893274	0,800625	0,122109	0,940655	0,909092	0,677094	0,663061	0,663061	0,892924

**s__Klebsiella_pneumoniae**	0,598712	0,909092	0,801001	0,903269	0,972822	0,940914	0,705439	0,749471	0,749471	0,882938
**s__Klebsiella_unclassified**	0,843407	0,873812	0,32546	0,903269	0,956117	0,825491	0,842131	0,74096	0,74096	0,871672
**s__Morganella_morganii**	0,554142	0,873812	0,133108	0,961352	0,843407	0,842131	0,843407	0,843407	0,843407	0,843407
**s__Proteus_mirabilis**	0,843407	0,843407	0,875137	0,961352	0,825491	0,875137	0,843407	0,825491	0,825491	0,902972
**s__Actinobacillus_unclassified**	0,827904	0,827904	0,843407	0,948064	0,883457	0,550269	0,714114	0,585515	0,585515	0,827904
**s__Aggregatibacter_segnis**	0,875137	0,873812	0,909092	0,961352	0,843407	0,842131	0,843407	0,843407	0,843407	0,967824
**s__Aggregatibacter_unclassified**	0,875137	0,542576	0,909092	0,961352	0,829492	0,826843	0,843407	0,843407	0,843407	0,977966
**s__Haemophilus_haemolyticus**	0,827904	0,937261	0,875137	0,940914	0,825491	0,940914	0,650123	0,532912	0,532912	0,776417
**s__Haemophilus_influenzae**	0,753299	0,843407	0,875137	0,961352	0,961352	0,92671	0,843407	0,825491	0,825491	0,843407
**s__Haemophilus_parahaemolyticus**	0,875137	0,431697	0,104407	0,961352	0,843407	0,694966	0,843407	0,843407	0,843407	0,843407
**s__Haemophilus_parainfluenzae**	0,566432	0,873812	0,532912	0,758119	0,82341	0,825491	0,934596	0,843407	0,843407	0,825491
**s__Haemophilus_paraphrohaemolyticus**	0,843407	0,843407	0,875137	0,961352	0,35625	0,405928	0,843407	0,825491	0,825491	0,825491
**s__Haemophilus_pittmaniae**	0,875137	0,873812	0,909092	0,961352	0,843407	0,792301	0,843407	0,847114	0,847114	0,753299
**s__Haemophilus_sputorum**	0,909092	0,95758	0,675334	0,930575	0,961897	0,809515	0,875137	0,843407	0,843407	0,843407
**s__Pseudomonas_unclassified**	0,875137	0,873812	0,909092	0,961352	0,843407	0,842131	0,843407	0,843407	0,843407	0,961352
**s__Akkermansia_muciniphila**	0,142634	0,824587	0,843407	0,843407	0,800687	0,89757	0,460361	0,340798	0,340798	0,843407
**s__Mulikevirus_unclassified**	0,875137	0,873812	0,148457	0,961352	0,800607	0,842131	0,478984	0,560708	0,560708	0,843407
**s__C2likevirus_unclassified**	0,806978	0,961352	0,940744	0,897976	0,908923	0,967085	0,705764	0,749471	0,749471	0,582098
**s__Lactococcus_phage_bIL67**	0,875137	0,873812	0,909092	0,961352	0,843407	0,875137	0,843407	0,843407	0,843407	0,903269
**s__Bacteroides_phage_B124_14**	0,875137	0,873812	0,909092	0,961352	0,843407	0,99275	0,843407	0,843407	0,843407	0,873812
**s__Lactobacillus_phage_Lc_Nu**	0,842131	0,825491	0,542576	0,961352	0,961352	0,439871	0,843407	0,825491	0,825491	0,875137
**s__Lactococcus_phage_936_sensu_lato**	0,875137	0,749471	0,909092	0,961352	0,843407	0,842131	0,843407	0,843407	0,843407	0,825491
**s__Lactococcus_phage_BM13**	0,875137	0,650123	0,909092	0,961352	0,537423	0,82317	0,843407	0,843407	0,843407	0,858615
**s__Lactococcus_phage_P680**	0,81479	0,852816	0,843407	0,948064	0,843407	0,880152	0,875137	0,843407	0,843407	0,961352
**s__Lactococcus_phage_phi7**	0,875137	0,749471	0,909092	0,961352	0,843407	0,842131	0,843407	0,843407	0,843407	0,825491

**s__Streptococcus_phage_ALQ13_2**	0,875137	0,873812	0,909092	0,961352	0,843407	0,842131	0,843407	0,875137	0,875137	0,825491
**s__Human_adenovirus_B**	0,875137	0,873812	0,909092	0,961352	0,843407	0,914984	0,843407	0,843407	0,843407	0,967824
**s__Human_adenovirus_D**	0,875137	0,873812	0,909092	0,961352	0,843407	0,842131	0,843407	0,843407	0,843407	0,74096
**s__Human_adenovirus_F**	0,875137	0,340433	0,909092	0,961352	0,598211	0,717285	0,843407	0,843407	0,843407	0,843407
